# The Molecular Epidemiology of the Highly Virulent ST93 Australian Community *Staphylococcus aureus* Strain

**DOI:** 10.1371/journal.pone.0043037

**Published:** 2012-08-10

**Authors:** Geoffrey W. Coombs, Richard V. Goering, Kyra Y. L. Chua, Stefan Monecke, Benjamin P. Howden, Timothy P. Stinear, Ralf Ehricht, Frances G. O’Brien, Keryn J. Christiansen

**Affiliations:** 1 Australian Collaborating Centre for Enterococcus and Sdtaphylococcus Species (ACCESS) Typing and Research, PathWest Laboratory Medicine – Western Australia, Royal Perth Hospital, Western Australia, Australia; 2 School of Biomedical Sciences, Curtin University, Western Australia, Australia; 3 Department of Medical Microbiology, Creighton University, Omaha, Nebraska, United States of America; 4 Department of Microbiology and Immunology, University of Melbourne, Victoria, Australia; 5 Austin Centre for Infection Research (ACIR), Infectious Diseases Department, Austin Health, Victoria, Australia; 6 Microbiology Department, Monash University, Victoria, Australia; 7 Department of Microbiology, Austin Health. Victoria, Australia; 8 Alere Technologies GmbH. Jena, Germany; 9 Institute for Medical Microbiology and Hygiene, Technical University of Dresden, Dresden, Germany; Rockefeller University, United States of America

## Abstract

In Australia the PVL - positive ST93-IV [2B], colloquially known as “Queensland CA-MRSA” has become the dominant CA-MRSA clone. First described in the early 2000s, ST93-IV [2B] is associated with skin and severe invasive infections including necrotizing pneumonia. A singleton by multilocus sequence typing (MLST) eBURST analysis ST93 is distinct from other *S aureus* clones. To determine if the increased prevalence of ST93-IV [2B] is due to the widespread transmission of a single strain of ST93-IV [2B] the genetic relatedness of 58 *S. aureus* ST93 isolated throughout Australia over an extended period were studied in detail using a variety of molecular methods including pulsed-field gel electrophoresis, *spa* typing, MLST, microarray DNA, SCC*mec* typing and *dru* typing. Identification of the phage harbouring the *lukS-PV/lukF-PV* Panton Valentine leucocidin genes, detection of allelic variations in *lukS-PV/lukF-PV*, and quantification of LukF-PV expression was also performed. Although ST93-IV [2B] is known to have an apparent enhanced clinical virulence, the isolates harboured few known virulence determinants. All PVL-positive isolates carried the PVL-encoding phage ΦSa2USA and the *lukS*-*PV/lukF*-*PV* genes had the same R variant SNP profile. The isolates produced similar expression levels of LukF-PV. Although multiple rearrangements of the *spa* sequence have occurred, the core genome in ST93 is very stable. The emergence of ST93-MRSA is due to independent acquisitions of different *dru*-defined type IV and type V SCC*mec* elements in several *spa-*defined ST93-MSSA backgrounds. Rearrangement of the *spa* sequence in ST93-MRSA has subsequently occurred in some of these strains. Although multiple ST93-MRSA strains were characterised, little genetic diversity was identified for most isolates, with PVL-positive ST93-IVa [2B]-t202-dt10 predominant across Australia. Whether ST93-IVa [2B] t202-dt10 arose from one PVL-positive ST93-MSSA-t202, or by independent acquisitions of SCCmec-IVa [2B]-dt10 into multiple PVL-positive ST93-MSSA-t202 strains is not known.

## Introduction

The community-associated methicillin resistant *Staphylococcus aureus* (CA-MRSA) worldwide epidemic is polyclonal, however several well characterized clones predominate in different regions of the world: Sequence type (ST) 8-IV [2B] (USA300) and ST1-IV [2B] (USA400) in North America [1], [2]; ST80-IV [2B] (European clone) in Europe [3], North Africa [4] and the Middle East [5]; ST59-V [5C2&5] (Taiwan clone) in Taiwan [6], ST30-IV [2B] (South West Pacific [SWP] CA-MRSA) in the Western Pacific [7], [8] and ST772-MRSA-V [5C2] (Bengal Bay clone) in India and Bangladesh [9]. Transmission of these clones into other regions has occurred [10], [11]. The occurrence of concurrent epidemics of CA-MRSA in many countries by different clones has been striking. Equally noteworthy are a number of common features of these epidemics, prominent among them the ability to cause severe infections in young otherwise healthy people and the carriage of *lukS-PV*/*lukF-PV* Panton Valentine Leukocidin (PVL) encoding genes by the organism.

In Australia the PVL - positive ST93-IV [2B], colloquially known as “Queensland CA-MRSA”, has recently emerged to become the dominant CA-MRSA clone. First described in the early 2000s, ST93 is a singleton by MLST eBURST analysis and is therefore distinct from other *S aureus* clones [12].

In the 2010 Australian Group for Antimicrobial Resistance (AGAR) Community *S aureus* Surveillance Programme (SAP10) ST93-IV [2B] accounted for 41.4% of all CA-MRSA, 27.6% of all MRSA and 4.9% of all *S aureus* community-onset infections (http://www.agargroup.org/files/FED%20REPORT%20SAP210%20MRSA%20FINAL%20shrink.pdf.). The mean age of patients with ST93-IV [2B] infections (31 years, median 25 years) was significantly lower (P<0.0001) than the mean age of patients with PVL negative CA-MRSA infections (53 years; median 57 years).

ST93-IV [2B] is associated with skin infection and severe invasive infection including necrotizing pneumonia, deep-seated abscess, osteomyelitis, septic arthritis and septicaemia [13], [14], [15]. Although ST93-IV [2B] has an apparent enhanced clinical virulence, the recently sequenced ST93-IV [2B] strain “JKD6159” has a relative paucity of recognizable virulence determinants [16], [17]. This strain however does contain genes encoding three important CA-MRSA virulence factors, Hla, PVL and α-type phenol soluble modulins (PSMs), and when compared to three other well-characterised Australian MRSA strains, ST1-IV [2B], ST30-IV [2B] and ST239-III [3B] and the epidemic North American strain, USA300, was shown to be the most virulent in two *in vivo* models [17].

While predominately an Australian strain, ST93-IV [2B] has been reported in New Zealand, accounting for 5.1% of all MRSA referred to the Institute of Environmental Science and Research in 2010 (http://www.surv.esr.cri.nz/PDF_surveillance/Antimicrobial/MRSA/aMRSA_2010.pdf), and in the United Kingdom, [18], where many cases have epidemiological links to Australia.

In Western Australia (WA) ST93-IV [2B] was first identified in 2003 [19] and in SAP10 accounted for 28.8% of the state’s CA-MRSA community-onset infections. In the mid 1990s *S aureus* screening of indigenous people living in WA remote communities demonstrated the most prevalent methicillin susceptible *S aureus* (MSSA) linage isolated was the PVL-positive ST93 MSSA clone [20]. Although seven CA-MRSA clones from genetically diverse backgrounds were identified in these communities, no ST93 MRSA was found during this time.

As Australia is a geographically large country with the majority of the population densely concentrated in a few major cities which are separated in many instances by vast desert areas, it is to be expected that different CA-MRSA clones will have evolved in different areas of Australia. To better understand the molecular epidemiology of ST93-IV [2B], the aim of this study was to analyse the genetic relatedness of *S. aureus* ST93 isolated throughout Australia over an extended period and to determine if the increased prevalence of ST93-IV [2B] has been due to the widespread transmission of a single strain of ST93-IV [2B] or has been due to multiple independent acquisitions of the SCC*mec* element into different strains of ST93 MSSA.

## Materials and Methods

### Bacterial Strains and Identification

Overall 58 ST93 *S. aureus* were included in the study. The 13 ST93-MSSA included four isolates from remote aboriginal communities in WA, isolated from 1995 to 2003; two isolates from the Northern Territory, isolated in 1992; five isolates from WA, isolated in 2008; and single isolates from Victoria, isolated in 2007, and Queensland, isolated in 2008. The 45 ST93-MRSA included 30 isolates from across Australia from the 2000 to 2008 AGAR Community onset *S. aureus* programs, and 15 isolates from WA from 2003 to 2009. *S. aureus* species and methicillin resistance was confirmed by the detection of *nuc* (thermostable extracellular nuclease) and *mecA* (methicillin resistance) genes by PCR as previously described [21].

### Susceptibility Testing

An antibiogram was performed by disk diffusion on Mueller-Hinton agar according to the Clinical and Laboratory Standards Institute (CLSI) recommendations [22]. A panel of eight antimicrobial drugs was tested: erythromycin (15 µg), tetracycline (30 µg), trimethoprim (5 µg), ciprofloxacin (5 µg), gentamicin (10 µg), rifampin (5 µg), fusidic acid (10 µg), and mupirocin (5 µg). CLSI interpretive criteria [23] were used for all drugs except fusidic acid [24] and mupirocin [25].

### PFG

Electrophoresis of chromosomal DNA was performed as previously described [26], using a contour-clamped homogeneous electric field (CHEF) DR III system (Bio-Rad Laboratories Pty Ltd). Chromosomal patterns were examined visually, scanned with a Quantity One device (Bio-Rad Laboratories Pty Ltd), and digitally analyzed using FPQuest (Bio-Rad Laboratories Pty Ltd). *S. aureus* strain NCTC 8325 was used as a reference strain.

### MLST and *Spa* Typing

Chromosomal DNA for MLST and *spa* typing was prepared using a DNeasy tissue kit (Qiagen Pty Ltd).

MLST was performed as previously described [27]. The sequences were submitted to http://www.mlst.net/where an allelic profile was generated and an ST assigned.

*spa* typing, a DNA sequenced-based analysis of the protein A gene variable region was performed as previously described [28] using the nomenclature as described on the Ridom website (http://spa.ridom.de/). Cluster analysis of *spa* sequences was performed using the spa typing plug-in tool of the BioNumerics software program (version 6.6; Applied Maths, Ghent, Belgium). The analysis compares and aligns sequences via an algorithm based on potential tandem *spa* repeat duplications, substitutions, and indels (the DSI model) [29]. A minimum spanning tree (MST) was generated from the similarity matrix with the root node assigned to the sequence type with the greatest number of related types. The default software parameters were used for analysis with a bin distance of 1.0%. Thus, the distance between *spa* types of 99% to 100% similarity was 0, 98% to 99% similarity was assigned a distance of 1, etc., on the MST. For cluster analysis, only *spa* types separated by an MST distance of ≤1 (i.e., if they were ≥98% similar) were considered closely related and assigned to the same cluster.

### DNA Microarray

Arrays and reagents were obtained from Alere Technologies, Jena Germany. The principle of the assay, related procedures, and a list of targets has been described previously [30], [31]. Target genes included species markers, markers for accessory gene regulator (*agr*) alleles and capsule types, virulence factors, resistance genes, staphylococcal superantigen-like/exotoxin-like genes (*set*/*ssl* genes) and genes encoding adhesion proteins and immune evasion factors. Probes for *mecA*, *ugpQ*, *xylR*, *kdp*, *ccr*’s, *mecI* and two probes for *mecR* were used for SCC*mec* typing.

### SCC*mec* Typing

The strategy used for SCC*mec* typing was as previously described [32]. SCC*mec* nomenclature is used as proposed by the International Working Group on the Classification of Staphylococcal Cassette Chromosome Elements (IWG-SCC) [33]. Briefly, the structural type is indicated by a Roman numeral, with a lowercase letter indicating the subtype, and the *ccr* complex and the *mec* complex are indicated by an Arabic numeral and an uppercase letter respectively in parenthesis. Where there is an extra *ccr* element, this is indicated by “&” and an Arabic numeral designating the *ccr* type. When there is an extra *ccr* element present whose precise location is unknown it is indicated by an “&” and *ccr* number outside the parentheses.

### PVL

PCR for the detection of PVL determinants was performed as previously described [34].

### PVL Phage Identification

PCRs were performed to detect the six PVL-encoding phages (ΦSa2MW, ΦSa2958, ΦPVL, Φ108PVL, ΦSLT and ΦSA2USA) as previously described [35], [36].

### Detection of Allelic Variations in *Luks-PV/lukF-PV* Genes

Detection of single nucleotide polymorphisms (SNPs) in a defined region of the *lukS-PV/lukF-PV* genes were performed as previously described [36], [37]. Sequences obtained were compared to the proposed progenitor PVL gene in ΦSLT/ST30.

### Quantification of *In vitro* LukF-PV Expression

PVL is a 2-component exotoxin and both LukS-PV and LukF-PV are required for activity. LukF-PV was measured instead of LukS-PV to obtain an anti-LukF-PV antibody with increased specificity of binding as there was more sequence divergence between *lukF-PV* and the orthologous 2-component *S. aureus* exotoxins compared to *lukS-PV*. To produce recombinant LukF-PV *lukF-PV* was PCR amplified using primers, forward 5′-CACCATGGCTCAACATATCACAC and reverse 5′-GCTCATAGGATTTTTTTCC. The resulting PCR product was then TOPO cloned into pENTR/SD/D-TOPO (Invitrogen). This plasmid was sequenced using M13 primers to confirm that the insert was present in the correct orientation without mutations. *lukF-PV* was subsequently cloned using an LR recombination reaction into the expression vector pET-DEST42 (Invitrogen) which introduced a C-terminal 6x-Histidine tag. This expression clone was used to transform Rosetta2 *E. coli* (Novagen). Soluble recombinant LukF-PV was produced by the Protein Production Unit, Monash University by growth of the expression strain in Auto Induction media at 28°C. The resulting recombinant LukF-PV was purified by Nickel purification followed by gel filtration and eluted in 100 mM NaP04, pH 7.4, 150 mM NaCl buffer. Aliquots were frozen and stored at −80°C. The concentration of recombinant LukF-PV was determined using the 2100 Bioanalyser P230 kit (Agilent).

### Quantification of LukF-PV Expression

Bacteria were grown in CCY media (3% yeast extract (Oxoid), 2% Bacto Casamino Acids (Difco), 2.3% sodium pyruvate (Sigma-Aldrich), 0.63% Na_2_HPO_4_, 0.041% KH_2_PO_4_, pH 6.7). Overnight cultures were diluted 1∶100 into fresh media and then incubated at 37°C with shaking (180 rpm) until stationary phase (OD600 ∼ 1.8). Culture supernatants were harvested by centrifugation and filter sterilized. The LukF-PV expression experiments were performed in at least duplicate for each *S. aureus* strain. Trichloroacetic acid was added to culture supernatants and incubated at 4°C overnight. Precipitates were then harvested by centrifugation, washed with acetone, air-dried and solubilized in a sample buffer containing 1.7% SDS and 1% 2-mercaptoethanol. The proteins were separated on 12% SDS-PAGE.

A peptide sequence specific to LukF-PV, HWIGNNYKDENRATHT was synthesized and HRP conjugated polyclonal chicken IgY raised against this peptide (Genscript). This antibody was used to detect LukF-PV with enhanced chemiluminescence. Images generated from the western blots were quantitated using GS800 Calibrated Densitometer and Quantity One (BioRad). 50 µg of recombinant LukF-PV was used as an internal standard on each gel, and was the positive control. Results observed with this standard were set to 1.0. All other results were shown as a ratio relative to this standard. RN4220 was used as a negative control.

### *Dru* Typing

Sequence analysis of the *mec*-associated *dru* region was performed as previously described [38]. A cluster analysis of *dru* sequences was performed using the Polymorphic VNTR plug-in tool of the BioNumerics software program (version 6.6; Applied Maths, Ghent, Belgium). The analysis compares and aligns sequences via an algorithm based on potential tandem *dru* repeat duplications, substitutions, and indels (the DSI model) [29]. A MST was generated from the similarity matrix with the root node assigned to the sequence type with the greatest number of related types. The default software parameters were used for analysis with a bin distance of 1.0%. Thus, the distance between *dru* types of 99% to 100% similarity was 0, 98% to 99% similarity was assigned a distance of 1, etc., on the MST. For cluster analysis, only *dru* types separated by an MST distance of ≤1 (i.e., if they were ≥98% similar) were considered closely related and assigned to the same cluster.

### Control Strain

The sequenced ST93-IVa [2B] strain JKD6159 (NCBI GenBank Accession No. CP002114 and CP002115) was included in this study for comparison [17].

## Results

Susceptibility results, SCC*mec* typing together with a summary of the resistance genes, *spa* types (using the Ridom Nomenclature) and *dru* type are shown in Table 1. Further characterisations are as follows:

**Table 1:** Characterisation of ST93 isolates.

Region	Reference Number	Year	Specimen	Antibiogram	DNA Microarray Resistance Genotype	PFGE	MLST	*spa* Sequence	*Spa* Type	SCC*mec* type	dru Sequence	*Dru* Type	*lukS*/*lukF PV*
**MSSA**													
NT	WBG 7735	1992	Unknown	Er^R^	*blaZ, blaI, blaR, ermC*	B	93	11-17-23-17-17-16-16-16-25	t4178	NA	NA		POSITIVE
NT	WBG 7762	1992	Unknown	Er^R^	*blaZ, blaI, blaR, ermC*	A		11-17-16-16-25	t4699	NA	NA		POSITIVE
Qld	UQ40	2008	Unknown		*blaZ, blaI, blaR*	A		11-17-23-17-17-16-16-25	t202	NA	NA		POSITIVE
Vic	DP 2039	2007	Unknown		*blaZ, blaI, blaR*	A	93	11-17-23-17-17-16-16-25	t202	NA	NA		POSITIVE
WA	C229T	2003	Throat		*blaZ, blaI, blaR*	A		11-17-23-17-17-16-16-25-25	t5767	NA	NA		POSITIVE
WA	N126W	2003	Hands	Er^R^	*blaZ, blaI, blaR, ermC*	C	93	11-17-23-17-17-16-16-25	t202	NA	NA		POSITIVE
WA	W17S	1995	Skin		*blaZ, blaI, blaR*	C	93	11-17-23-17-17-16-16-25	t202	NA	NA		POSITIVE
WA	Y113S	1996	Skin	Er^R^	*blaZ, blaI, blaR, ermC*	C		11-17-23-17-17-16-16-25	t202	NA	NA		POSITIVE
WA	9506160A	2008	Blood		*blaZ, blaI, blaR*	A		11-17-23-17-17-16-16-25	t202	NA	NA		POSITIVE
WA	9509712N	2008	Pleural Fl		*blaZ, blaI, blaR*	A		04-23-17-17-16-16-25	t6485	NA	NA		POSITIVE
WA	9524093R	2008	Buttock		*blaZ, blaI, blaR*	A		11-17-23-17-17-16-16-25	t202	NA	NA		POSITIVE
WA	9525206A	2008	Blood		*blaZ, blaI, blaR*	A		11-17-23-17-17-16-16-25	t202	NA	NA		POSITIVE
WA	9529120L	2008	Heel	Er^R^	*blaZ, blaI, blaR, ermC*	A		11-17-23-17-17-16-16-25-25	t5767	NA	NA		POSITIVE
**MRSA**													
ACT	SAPTCH92	2000	Blood	Ox^R^	*mecA, blaZ, blaI, blaR*	D		11-17-23-17-17-16-16-25	t202	IVa [2B]	5a-2d-4a-0-2d-5b-3a-2g-3b-4e	dt10a	POSITIVE
ACT	SAPTCH53	2008	Boil	Ox^R^	*mecA, blaZ, blaI, blaR*	D		11-17-23-17-17-16-16-25	t202	IVa [2B]	5a-2d-4a-0-2d-5b-3a-2g-3b-4e	dt10a	POSITIVE
NSW	SAPRPAH96	2000	Eye	Ox^R^ Er^R^	*mecA, blaZ, blaI, blaR, ermC*	D		11-17-23-17-17-16-16-25	t202	IVa [2B]	5a-2d-3b	dt3b	POSITIVE
NSW	SAPWH23	2000	Wound	Ox^R^	*mecA, blaZ, blaI, blaR*	D		11-17-23-17-17-16-16-25	t202	IVa [2B]	5a-2d-3b-4e	dt4d	POSITIVE
NSW	SAPWH39	2000	Wound	Ox^R^ Er^R^	*mecA, blaZ, blaI, blaR*	D		11-17-23-17-17-16-16-25	t202	IVa [2B]	5a-2d-3b-4e	dt4d	POSITIVE
NSW	SAPWH61	2000	Wound	Ox^R^	*mecA, blaZ, blaI, blaR*	D		11-17-23-17-17-16-16-25	t202	IVa [2B]	5a-2d-4a-0-2d-5b-3a-2g-3b-4e	dt10a	POSITIVE
NSW	SAPWH64	2000	Wound	Ox^R^	*mecA, blaZ, blaI, blaR, ermC*	E	93	11-17-23-17-17-16-16-25	t202	IVa [2B]	5a-2d-4a-0-2d-5b-3a-2g-3b-4e	dt10a	POSITIVE
NSW	SAPWH94	2000	Wound	Ox^R^	*mecA, blaZ, blaI, blaR*	D		11-17-23-17-17-16-16-25	t202	IVa [2B]	5a-2d-4a-0-2d-5b-3a-2g-3b-4e	dt10a	POSITIVE
NSW	SAPWH71	2004	Blood	Ox^R^	*mecA, blaZ, blaI, blaR*	I	93	11-17-23-17-17-16-16	t6487	IVa [2B]	5a-2d-4a-0-2d-5b-3a-2g-3b-4e	dt10a	NEGATIVE
NSW	SAPCRGH95	2007	Blood	Ox^R^	*mecA, blaZ, blaI, blaR*	D		11-17-23-17-17-16-16-25	t202	IVa [2B]	5a-2d-4a-0-2d-5b-2a-2g-3b-4e	dt10g	POSITIVE
NSW	SAPRPAH21	2007	Sputum	Ox^R^	*mecA, blaZ, blaI, blaR*	D		11-17-23-17-17-16-16-25	t202	IVa [2B]	5a-2d-4a-0-2d-5b-3a-2g-3b-4e	dt10a	POSITIVE
NSW	SAPRPAH7	2008	Ulcer	Ox^R^	*mecA, blaZ, blaI, blaR, ermC*	D		11-17-23-17-17-16-16-25	t202	IVa [2B]	5a-2d-4a-0-2d-5b-3a-2g-3b-4e	dt10a	POSITIVE
													POSITIVE
NSW	SAPWH10	2008	Wound	Ox^R^	*mecA, blaZ, blaI, blaR*	D		11-17-23-17-17-16-16-25	t202	IVa [2B]	5a-2d-4a-0-2d-5b-3a-2g-3b-4e	dt10a	POSITIVE
													POSITIVE
NSW	SAPWH53	2008	Wound	Ox^R^ Er^R^ Tmp^R^	*mecA, blaZ, blaI, blaR, msr(A)*	K	93	11-17-17-16-16-25	t1811	IVa [2B]	5a-2d-3b	dt3b	NEGATIVE
NT	SAPRDH61	2006	Wound	Ox^R^	*mecA, blaZ, blaI, blaR*	D		11-17-23-17-17-16-16-25	t202	IVa [2B]	5a-2d-4a-0-2d-5b-3a-2g-3b-4e	dt10a	POSITIVE
NT	SAPRDH27	2007	Thigh	Ox^R^	*mecA, blaZ, blaI, blaR*	D		11-17-23-17-17-16-16-25	t202	IVa [2B]	5a-2d-4a-0-2d-5b-3a-2g-3b-4e	dt10a	POSITIVE
NT	SAPRDH2	2008	Leg	Ox^R^	*mecA, blaZ, blaI, blaR*	D		11-17-23-17-17-16-16-25	t202	IVa [2B]	5a-2d-4a-0-2d-5b-3a-2g-3b-4e	dt10a	POSITIVE
Qld	SAPRBH98	2000	Leg	Ox^R^	*mecA, blaZ, blaI, blaR*	D		11-17-23-17-17-16-16-25	t202	IVa [2B]	5a-2d-4a-0-2d-5b-3a-2g-3b-4e	dt10a	POSITIVE
Qld	SAPRBH12	2005	Blood	Ox^R^	*mecA, blaZ, blaI, blaR*	D		11-17-23-17-17-16-16-25	t202	IVa [2B]	5a-2d-4a-0-2d-5b-3a-2g-3b-4e	dt10a	POSITIVE
Qld	SAPGCH3	2006	Abscess	Ox^R^	*mecA, blaZ, blaI, blaR*	D		11-17-23-17-17-16-16-25	t202	IVa [2B]	5a-2d-4a-0-2d-5b-3a-2g-3b-4e	dt10a	POSITIVE
Qld	SAPRBH14	2006	Wound	Ox^R^	*mecA, blaZ, blaI, blaR*	D		11-17-23-17-17-16-16-25	t202	IVa [2B]	5a-2d-4a-0-2d-5b-3a-2g-3b-4e	dt10a	POSITIVE
Qld	SAPCBH10	2008	Aspirate	Ox^R^	*mecA, blaZ, blaI, blaR*	D		11-17-23-17-17-16-16-25	t202	IVa [2B]	5a-2d-4a-0-2d-5b-3a-2g-3b-4e	dt10a	POSITIVE
Qld	SAPGCH28	2008	Foot	Ox^R^	*mecA, blaZ, blaI, blaR*	D		11-17-23-17-17-16-16-25	t202	IVa [2B]	5a-2d-4a-0-2d-5b-3a-2g-3b-4e	dt10a	POSITIVE
Qld	SAPRBH1	2008	Forearm	Ox^R^	*mecA, blaZ, blaI, blaR*	D		11-17-23-17-17-16-16-25	t202	IVa [2B]	5a-2d-4a-0-2d-5b-3a-2g-3b-4e	dt10a	POSITIVE
SA	SAPGPSA73	2000	Unknown	Ox^R^	*mecA, blaZ, blaI, blaR*	D		11-17-23-17-17-16-16-25	t202	IVa [2B]	5a-2d-4a-0-2d-5b-3a-2g-3b-4e	dt10a	POSITIVE
SA	SAPIMVS24	2006	Abscess	Ox^R^	*mecA, blaZ, blaI, blaR*	J		11-17-23-17-17-16-16-25	t202	IVa [2B]	5a-2d-4a-0-2d-5b-3a-2g-3b-4e	dt10a	POSITIVE
SA	SAPIMVS31	2008	Boil	Ox^R^	*mecA, blaZ, blaI, blaR*	D		11-17-23-17-17-16-16-25	t202	IVa [2B]	5a-2d-4a-0-2d-5b-3a-2g-3b-4e	dt10a	POSITIVE
Vic	SAPRCH74	2000	Wound	Ox^R^	*mecA, blaZ, blaI, blaR*	D		11-17-23-17-17-16-16-25	t202	IVa [2B]	5a-2d-3b-4e	dt4d	POSITIVE
Vic	SAPAH21	2008	Wound	Ox^R^ Te^R^	*mecA, blaZ, blaI, blaR, tetK*	D		11-17-23-17-17-16-16-16-25	t4178	IVa [2B]	5a-2d-4a-0-2d-5b-3a-2g-3b-4e	dt10a	POSITIVE
WA	16790	2003	Axilla	Ox^R^	*mecA, blaZ, blaI, blaR*	D		11-17-23-17-17-16-16-25	t202	IVa [2B]	5a-2d-4a-0-2d-5b-3a-2g-3b-4e	dt10a	POSITIVE
WA	16815	2005	Abscess	Ox^R^	*mecA, blaZ, blaI, blaR*	D		11-17-23-17-17-16-16-25	t202	IVa [2B]	5a-2d-4a-0-2d-5b-3a-2g-3b-4e	dt10a	POSITIVE
WA	15586	2008	Lip	Ox^R^	*mecA, blaZ, blaI, blaR*	D		11-17-23-17-17-16-16-25	t202	IVa [2B]	5a-2d-3b	dt3b	POSITIVE
WA	15587	2008	Nose	Ox^R^ Er^R^	*mecA, blaZ, blaI, blaR, ermC*	D		11-17-23-17-17-16-16-25	t202	IVa [2B]	5a-2d-4a-0-2d-5b-3a-2g-3b-4e	dt10a	POSITIVE
WA	16414	2008	Thigh	Ox^R^	*mecA, blaZ, blaI, blaR*	D		11-17-23-17-17-16-16-25	t202	IVa [2B]	5a-2d-3b	dt3b	POSITIVE
WA	16475	2008	ETT	Ox^R^	*mecA, blaZ, blaI, blaR*	D		11-17-23-17-17-16-16-25	t202	IVa [2B]	5a-2d-4a-0-2d-4f-3a-2g-3b-4e	dt10i	POSITIVE
WA	17164	2008	Abscess	Ox^R^	*mecA, blaZ, blaI, blaR*	J		11-17-23-17-17-16-16-25	t202	IVa [2B]	5a-2d-4a-0-2d-5b-3a-2g-3b-4e	dt10a	POSITIVE
WA	18158	2008	Wound	Ox^R^	*mecA, blaZ, blaI, blaR*	D		11-17-23-17-17-16-16-25	t202	IVa [2B]	5a-2d-3b	dt3b	POSITIVE
WA	18385	2008	Shin	Ox^R^	*mecA, blaZ, blaI, blaR*	D		11-17-23-17-17-16-16-25	t202	IVa [2B]	5a-2d-4a-0-2d-5b-3a-2g-3b-4e	dt10a	POSITIVE
WA	18418	2008	Nose	Ox^R^	*mecA, blaZ, blaI, blaR*	D		11-17-23-17-17-16-16-25	t202	IVa [2B]	5a-2d-3b	dt3b	POSITIVE
WA	20198	2008	Nose	Ox^R^	*mecA, blaZ, blaI, blaR*	H	93	11-17-23-17-17-16-16-25	t202	V [5C2&5]	5a-2d-4a-0-2d-5b-3a-2g-1c-4e-3e	dt11i	NEGATIVE
WA	SAPRPH48	2008	Wound	Ox^R^	*mecA, blaZ, blaI, blaR*	J	93	11-17-23-17-17-16-16-25	t202	IVa [2B]	5a-2d-4a-0-2d-5b-3a-2g-3b-4e	dt10a	POSITIVE
WA	16908	2009	Leg	Ox^R^	*mecA, blaZ, blaI, blaR, (dfrA)*	F	93	11-17-23-17-17-16-16-25	t202	IVa [2B]	5a-2d-4a-0-2d-5b-3a-2g-3b-4e	dt10a	POSITIVE
WA	17090	2009	Wound	Ox^R^	*mecA, blaZ, blaI, blaR, qacC*	G	93	11-17-23-17-17-16-16-25	t202	IVa [2B]	5a-2d-4a-0-2d-5b-3a-2g-3b-4e	dt10a	POSITIVE
WA	17195	2009	Nose	Ox^R^ Er^R^	*mecA, blaZ, blaI, blaR, ermC, qacC*	D	93	11-17-23-17-17-16-16-25	t202	IVa [2B]	5a-2d-4a-0-2d-5b-3a-2g-3b-4e	dt10a	POSITIVE
WA	20548	2009	Abscess	Ox^R^	*mecA, blaZ, blaI, blaR*	D		11-17-23-17-17-16-16-25	t202	IVa [2B]	5a-2d-4a-0-2d-5b-3a-2g-3b-4e	dt10a	POSITIVE
**Control Strain**													
Vic	JKD6159	2004	Blood	Ox^R^	*mecA, blaZ, blaI, blaR*	D	93	11-17-23-17-17-16-16-25	t202	IVa [2B]	5a-2d-4a-0-2d-5b-3a-2g-3b-4e	dt10a	POSITIVE

Regions: ACT, Australian Capital Territory; NSW, New South Wales; NT, Northern Territory, Qld, Queensland; SA, South Australia; Vic, Victoria; WA, Western Australia.

Antibiogram: Ox, oxacillin; Em, erythromycin; Te, tetracycline.

Resistance Genotype: *mecA*, alternate penicillin binding protein 2 gene; *blaZ*, beta lactamase gene; *blaI*, beta-lactamase repressor (inhibitor) gene; *blaR* beta-lactamase regulatory protein gene, *ermC*, erythromycin/clindamycin resistance gene; *msr(A)*, macrolide resistance gene; *tetK*, tetracycline resistance gene; *dfrA*, trimethoprim resistance gene; qacC quaternary ammonium compound resistance gene protein C.

PFGE, pulsed field gel electrophoresis; MLST, multilocus sequence type; SCCmec, staphylococcal cassette chromosome mec; *spa, Staphylococcus aureus* protein gene A; *dru*, the direct-repeat unit (*dru*) variable-number tandem repeat region adjacent to IS431 in SCC*mec.*

*lukF*/*lukS* PV, Panton Valentine leucocidin F and S component genes.

### Molecular Typing

**Figure 1:**
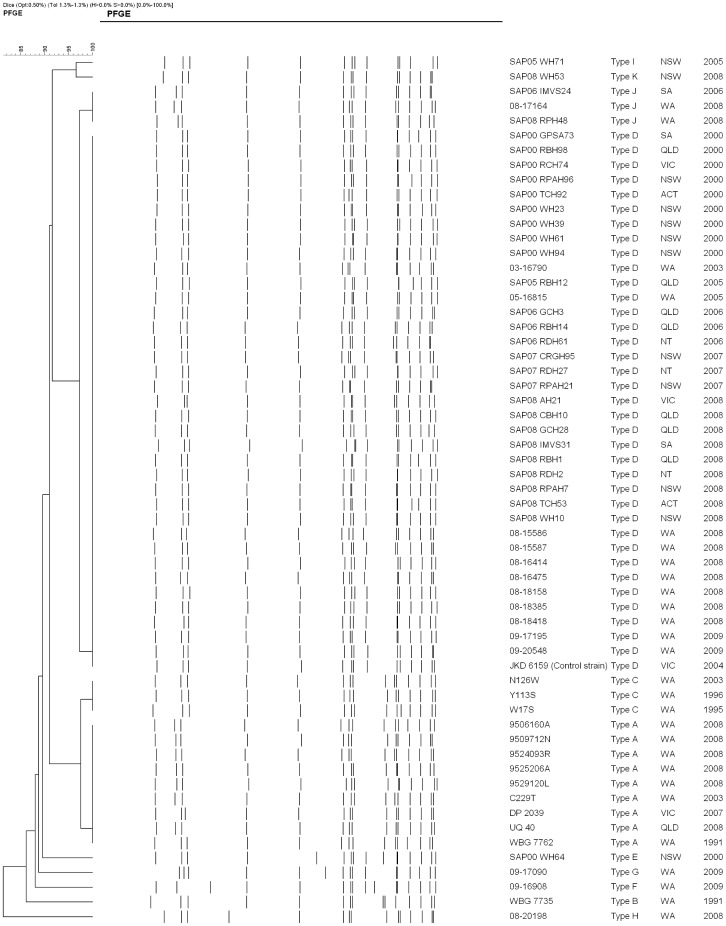
Dendrogram of the 58 pulsed-field gel electrophoresis patterns (PFGE) of ST93 (13 MSSA and 45 MRSA). Sequenced JKD6159 strain was used as the ST93 control. *S. aureus* strain NCTC 8325 was used as the reference strain.

By PFGE the 58 isolates (13 MSSA and 45 MRSA) had ≥80% similarity with the sequenced JKD6159 strain (Figure 1). Eleven pulsotypes were identified. The MSSA isolates consisted of three pulsotypes, “A” – “C” with 12 of the 13 isolates grouped into two closely related pulsotypes; “A” (9 isolates) and “C” (3 isolates). The MRSA isolates consisted of eight pulsotypes (“C” – “K”) with 39 of the 45 isolates grouped into two closely related pulsotypes; “D” (36 isolates) and “J” (3 isolates). The MSSA pulsotypes “A” and “C” and the MRSA pulsotypes “D” and “J” were 92% related; the difference presumably due to the insertion of the SCC*mec* type IVa [2B] element into an existing restriction fragment in the two MRSA pulsotypes. Single isolates of closely related MRSA pulsotypes “I” (SAPWH71) and “K” (SAPWH53) lacked a PVL-encoding phage. MRSA pulsotype “H” (20198) also lacked a PVL-encoding phage, however unlike the other MRSA, carried the SCC*mec* type V element with an additional *ccr* element [5C2&5]. The remaining MSSA and three MRSA isolates were classified into four unique pulsotypes (pulsotypes B, E, F, G).

Isolates representing each pulsotype were identified as ST93 by MLST.

**Figure 2:**
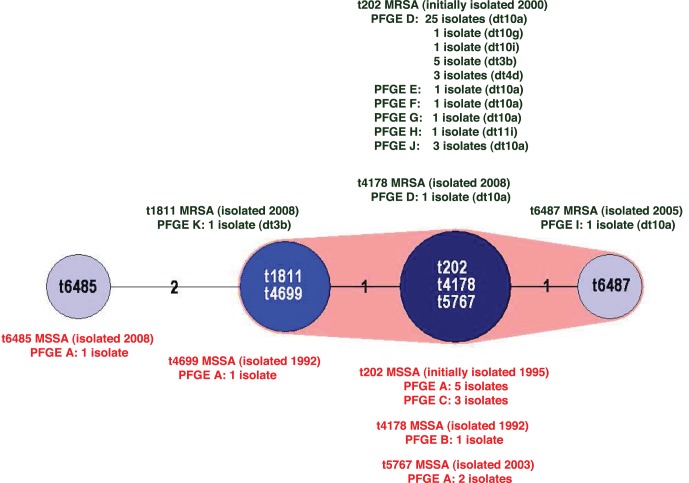
Minimum spanning tree (MST) of the seven ST93 *spa* types. Cluster analysis was performed using the spa typing plug-in tool of the BioNumerics program. *spa* types separated by an MST distance of ≤1 (i.e., if they were ≥98% similar) were considered closely related and assigned to the same cluster. MSSA and MRSA *spa* types are designated in red and green print respectively. Pulsed-field gel electrophoresis (PFGE) pulsotypes and *dru* types (dt) are recorded for each *spa* type.

Seven *spa* types were identified with the majority of isolates characterised as t202 (8/13 MSSA and 42/45 MRSA). The MST algorithm clustered the *spa* types into two significantly different groups; t202, t4178, t5767; t1811, t4699; plus t6487; and t6485 (Figure 2).

### DNA Microarray

The β-lactamase operon (*bla*Z, *blaI*, *blaR*) was detected in all isolates (Table 1). Apart from isolate 20198, the MRSA carried *mecA* as a part of the SCC*mec* type IVa [2B] element. Carriage of other resistance genes was infrequent and variable. Five of the MSSA were phenotypically erythromycin resistant and carried *ermC*. Of the five erythromycin resistant MRSA isolates, three carried *ermC* and one the staphylococcal *msr(A)* macrolide efflux protein gene. A macrolide resistance gene was not detected in one isolate that demonstrated phenotypic resistance (SAPWH39). Two *ermC* harbouring MRSA isolates were not phenotypically erythromycin resistant. A single MRSA isolate harboured the *tetK* tetracycline resistant gene (isolated in Victoria in 2008), and two MRSA isolates carried the quaternary ammonium compound resistance protein C (*qacC*) gene (isolated in WA in 2009).

The 58 isolates were *agr* group III and capsule type 8. Although the enterotoxin and *tst1* genes were absent from all isolates, the enterotoxin homologue ORF CM14 was present in 34 isolates (4 MSSA and 30 MRSA) (Table S1). All isolates carried the *hlb*, *hld* and *hlIII* hemolysin genes; the staphylokinase (*sak*), chemotaxis inhibitory protein (*chp*) and staphylococcal complement inhibitor (*scn*) genes; and the *aur*, *splA*, *sspA*, *sspB*, *sspP* protease genes. Although the gene for a biofilm-associated protein, *bap*, was absent, the biofilm operon icaACD was present in all isolates. Most isolates carried the leukocidin *lukX* and *lukY* genes, the *hl*, *hla* hemolysin genes and the *splE* protease genes.

The staphylococcal superantigen like or exotoxin-like genes (*set* or *ssl* genes) and genes encoding MSCRAMMS (microbial surface components recognizing adhesive matrix molecules) and the immune evasion factors were homogeneous and characteristic for ST93 (Tables S2, S3, S4 and S5).

### Panton Valentine Leukocidin (PVL)

Apart from three MRSA isolates (SAPWH71, SAPWH53 and 20198) the *lukS*-*PV/lukF*-*PV* genes were detected in all isolates by array hybridisation and PCR. All *lukS*-*PV/lukF*-*PV* positive isolates carried the PVL-encoding phage ΦSa2USA. Using the proposed progenitor PVL gene in ΦSLT/ST30 as a reference sequence, all isolates were similar, having the same R variant SNP profile with three substitutions compared to ΦSLT. This SNP profile is associated with the ΦSa2USA phage.

**Figure 3:**
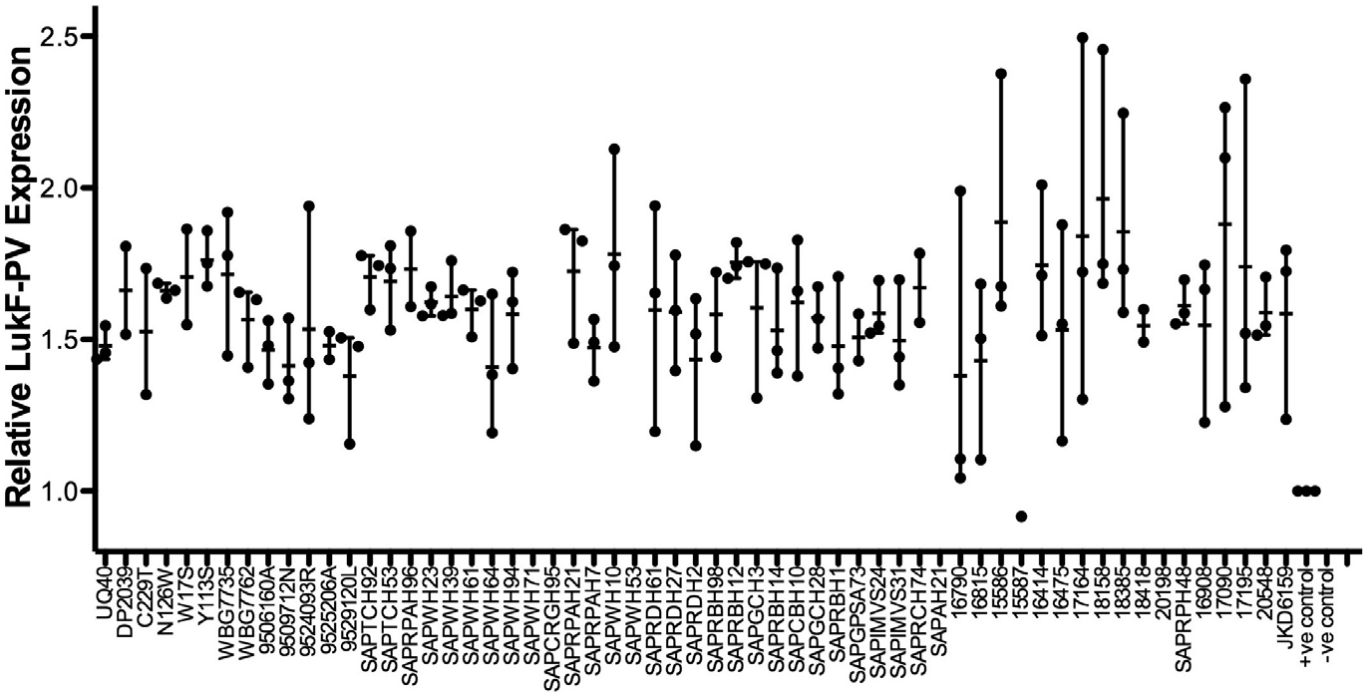
Relative LukF-PV expression in ST93 isolates. All isolates were tested for LukF-PV expression using western blot and LukF-PV specific antibody. Results are expressed as optical density of test strain relative to a 50 µg control of rLukF-PV that was run on every gel. All experiments were performed with multiple replicates and mean and range is shown. Positive control, rLUKF-PV; negative control, RN4220.

lukF-PV expression is shown in Figure 3 and was measured to determine if there was a consistent expression profile across different ST93 strains. As expected, ST93 isolates which did not contain *lukS*-*PV/lukF*-*PV* did not express LukF-PV. However, there were three isolates (SAPCRGH95 isolated in NSW, SAPAH21 isolated in Vic, and 15587 isolated in WA) which were PVL positive by array hybridization and PCR but did not express LukF-PV indicating that there may be regulatory differences such as an *agr* defect in these isolates to account for the absence of LukF-PV. All other isolates produced LukF-PV, with expression levels similar between most strains.

### *Dru* Typing

**Figure 4:**
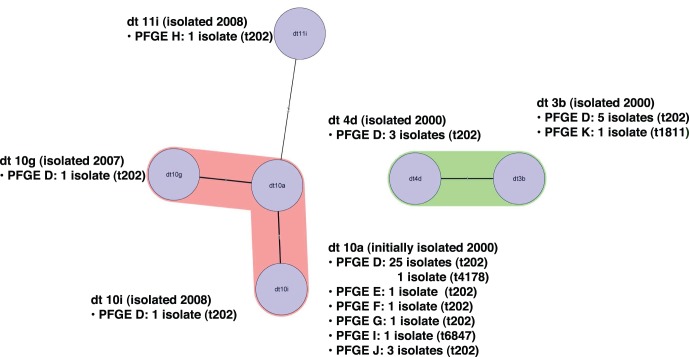
Minimum spanning tree (MST) of the six ST93 *dru* types. Cluster analysis was performed using the Polymorphic VNTR plug-in tool of the BioNumerics program. *dru* types separated by an MST distance of ≤1 (i.e., if they were ≥98% similar) were considered closely related and assigned to the same cluster. Pulsed-field gel electrophoresis (PFGE) pulsotypes and *spa* types are recorded for each *dru* type.

Six *dru* types were identified (Table 1). The majority of isolates (35/45) were dt10 (33 dt10a, 1 dt10 g and 1 dt10i). The remaining nine SCC*mec* type IVa [2B] isolates were *dru* type dt4d (three isolates) and dt3b (6 isolates). The SCC*mec* type V [5C2&5] isolate (20198) was dt11i. The MST algorithm clustered the six *dru* types into three significantly different groups; dt10, dt4d plus dt3b, and dt11 (Figure 4). This suggests the SCC*mec* element may have been acquired on at least three occasions by ST93.

## Discussion

CA-MRSA is thought to emerge when a locally prevalent strain of methicillin susceptible *S. aureus* (MSSA) acquires a SCC*mec* element and utilizes mobile genetic elements and single nucleotide polymorphisms to establish local and geographic niches [39]. Although the vertical and horizontal transmission of SCC*mec* elements into *S. aureus* has occurred on multiple occasions in the Australian community only a small number of clones have successfully found an ecological niche to predominate over other CA-MRSA clones [40]. PVL-positive ST93-IV [2B] is one such clone, and since 2000 has been reported across Australia and is responsible for the increasing prevalence of CA-MRSA infections nationwide [13].

Conflicting hypotheses have been proposed to explain the molecular evolution of ST93-MRSA. In 2008 Munckhof and colleagues found little genetic diversity within ST93-IV [2B] suggesting it arose from one PVL-positive binary subtype of ST93 MSSA after the acquisition of SCC*mec* [41]. However in 2009 Tong and colleagues identified multiple *spa* types in ST93-MRSA and ST93-MSSA and proposed their data supported an early acquisition of SCC*mec* with subsequent rearrangement of the *spa* sequence or multiple independent acquisitions of SCC*mec* and coexistence of MSSA and MRSA versions of the same lineage [42]. Although seven *spa* types were described in this study, cluster analysis of the seven *spa* sequences using the Spa typing plug-in tool of the BioNumerics software program shows six of the *spa* types are closely related and can be assigned to a single cluster (data not shown).

Unlike the Tong study, which examined the *spa* types of geographically localized ST93 *S aureus* collected over a short period, the current study examined ST93 *S aureus* isolated across Australia over sixteen years using a variety of molecular tools, providing greater power to detect unique evolutionary events in geographically diverse regions.

Prior to the isolation of ST93-IV [2B], *S aureus* surveillance screening of aboriginal people living in 11 remote Western Australian communities identified ST93 as the most prevalent MSSA lineage [20]. Although located in three geographically distant regions of WA, the ST93-MSSA examined from these communities, (W17S isolated in 1995, Y113S in 1996 and C229T and N126W in 2003, and) exhibit limited diversity within their PFGE patterns, *spa* types and microarray DNA profiles. Their two *spa* types, t202 [3 isolates (“PFGE C”)] and t5767 (“PFGE A”) are closely related and are assigned to the same cluster. The microarray DNA profiles for the two ST93-MSSA isolated in the Northern Territory in 1992 (WBG7735 and WBG7762) are homogeneous with the four WA remote community ST-93 MSSA. In addition their PFGE patterns are either identical (“PFGE A”) or 90% related (“PFGE B”), and their *spa* types, t4699 and t4178, are assigned to the same cluster. The DNA microarray profiles for the five ST93-MSSA, (9506160A, 9509712N, 9524093R, 9525206A and 9529120L) isolated in the state’s capital, Perth in 2008 (located 700–2000 km from the remote communities and over 3,000 km from the Northern Territory border) are also homogeneous with the Western Australian remote community strains. The PFGE pattern for these isolates is “PFGE A”. The *spa* types for four of these strains are t202 (3 isolates) and t5767. The *spa* type for 9509712N (t6485) cannot be assigned to the same cluster. The PFGE patterns, *spa* types and microarray DNA profiles for the MSSA isolated on the Australian eastern seaboard (UQ40– Queensland in 2008 and DP2039– Victoria in 2007) are identical to three Perth ST93-MSSA-t202 isolates.

As shown in Figure 1 the MRSA isolates are ≥80% related by PFGE with the majority of isolates falling into pulsotype D. Similar to the MSSA pulsotypes, pulsotype D was dispersed throughout Australia over the eight years. Although rearrangement of the *spa* sequence has occurred several times, the PFGE patterns and microarray DNA profiles of the 13 ST-93 MSSA isolates suggests the ST93 core and accessory genome is very stable. All carry the PVL-encoding phage ΦSa2USA and their *lukS*-*PV/lukF*-*PV* genes have the same R variant SNP profile. The isolates produce similar expression levels of LukF-PV with no apparent relationship between subtype and PVL expression. The emergence of five different *spa* types, albeit four types assigned to the same cluster, suggests ST93-MSSA emerged some time ago from a common *spa* type. As the *spa* sequences are similar it is not possible to predict the ancestral strain; however one strain, ST93-MSSA-t202, predominates and has successfully disseminated across Australia.

Like ST93-MSSA, ST93-MRSA has multiple *spa* types; including the closely related t202 and t4178, identified in ST93-MSSA, t1811 and t6487, all of which are assigned to the same cluster. t202 has the largest number of isolates; 42 of the 45 ST93-MRSA. SCC*mec* and *dru* typing indicates the SCC*mec* element has been acquired by ST93-MRSA-t202 on at least three occasions; dt10 (SCC*mec* type IVa [2B]), dt3b/dt4d (SCC*mec* type IVa [2B]) and dt11i (SCC*mec* type V [5C&5]). Unlike ST93-IVa [2B]-t202, ST93-V [5C&5]-t202 does not carry the *lukS-PV*/*lukF*-*PV* genes. The PVL-negative ST93-IVa [2B]-t1811 isolate may have arisen by independent acquisition of SCC*mec* IVa [2B] or by the subsequent rearrangement of the *spa* sequence.

As for ST93-MSSA, the PFGE patterns and microarray DNA profiles of the 45 ST-93 MRSA isolates suggests the ST93 core and accessory genome is stable. Forty three of the 45 isolates carry the PVL-encoding phage ΦSa2USA. The *lukS*-*PV/lukF*-*PV* genes have the same R variant SNP profile and produce similar expression levels of LukF-PV as reported in ST93-MSSA.

Apart from the *ermC* gene which was identified in several early ST93-MSSA and ST93-MRSA isolates, ST93 *S. aureus* initially carried few antibiotic resistance elements. However since 2008, in addition to *mecA* and *ermC*, some isolates of ST93-MRSA have acquired the *msr(A)* and *tetK* resistance genes. Although the *dfrA* gene was not detected by the microarray DNA, SAPWH53 is phenotypically trimethoprim resistant (presumably due to an alternative trimethoprim resistance gene or a different *dfrA* allele). In addition, the quaternary ammonium compound resistance protein C gene *qacC* is carried by two isolates. The acquisition of several resistance genes by an epidemic PVL-positive CA-MRSA clone is not unique to ST93-IV [2B]. The USA300 clone (ST8-IV [2B]), initially resistant only to semi-synthetic penicillins and macrolides, is now, frequently resistant to other antimicrobial agents including clindamycin, tetracycline, mupirocin, and the fluoroquinolones; occasionally resistant to gentamicin and trimethoprim-sulfamethoxazole, and may have reduced susceptibility to daptomycin [43].

Single strain outbreaks of ST93-IV [2B] have not been reported in Australian hospitals, however as has been reported in United States hospitals with USA300 [44], ST93-IV [2B] has become a major cause of healthcare-associated/onset infection. In 2008 Munckhof and colleagues reported nearly three quarters of nmMRSA infections in their hospital-based study were healthcare associated, of which ST93-IV [2B] predominated [41].

### Conclusion

This study has demonstrated that although multiple rearrangements of the *spa* sequence have occurred, the core genome in ST93 *S. aureus* is very stable. Since 2008 PVL-positive ST93-MSSA-t202 has become the predominant ST93-MSSA across Australia. We have shown the emergence of ST93-MRSA has been due to independent acquisitions of different *dru*-defined type IV and type V SCC*mec* elements in several *spa-*defined ST93-MSSA backgrounds. Rearrangement of the *spa* sequence in ST93-MRSA has subsequently occurred in some of these strains. Although several ST93-MRSA strains have been identified in this study, little genetic diversity was identified for most MRSA isolates, with PVL-positive ST93-IVa [2B]-t202-dt10 predominant across Australia. However to determine if ST93-IVa [2B] t202-dt10 has arisen from one PVL-positive ST93-MSSA-t202, or by independent acquisitions of SCCmec-IVa [2B]-dt10 into multiple PVL-positive ST93-MSSA-t202 strains will require whole genomic sequencing of the isolates. Furthermore, comparative genomic sequencing may further enhance our understanding of the molecular basis for the emergence and increased virulence of ST93 CA-MRSA. At a time when this clone is acquiring additional resistance genes and an increased potential for infections in the healthcare setting, understanding the means for SCC*mec* acquisition, virulence determinants and transmission dynamics is crucial if we are to prevent this clone from becoming established in hospitals.

## Supporting Information

Table S1 **Microarray DNA ST93 virulence profile**
(DOCX)

Table S2 **Microarray DNA ST93 ST93 staphylococcal superantigen/enterotoxin-like genes (set/ssl) profile.**
(DOCX)

Table S3 and S4 **Microarray DNA ST93 ST93 MSCRAMMs and adhesion profile.**
(DOCX)

Table S5 **Microarray DNA ST93 immunevasion and miscellaneous profile.**
(DOCX)

## References

[1] Tenover FC, McDougal LK, Goering RV, Killgore G, Projan SJ, et al (2006) Characterization of a strain of community-associated methicillin-resistant Staphylococcus aureus widely disseminated in the United States. J Clin Microbiol 44: 108–118. 16390957 10.1128/JCM.44.1.108-118.2006 PMC1351972

[2] McDougal LK, Steward CD, Killgore GE, Chaitram JM, McAllister SK, et al (2003) Pulsed-field gel electrophoresis typing of oxacillin-resistant Staphylococcus aureus isolates from the United States: establishing a national database. J Clin Microbiol 41: 5113–5120. 14605147 10.1128/JCM.41.11.5113-5120.2003 PMC262524

[3] Vandenesch F, Naimi T, Enright MC, Lina G, Nimmo GR, et al (2003) Community-acquired methicillin-resistant Staphylococcus aureus carrying Panton-Valentine leukocidin genes: worldwide emergence. Emerg Infect Dis 9: 978–984. 12967497 10.3201/eid0908.030089 PMC3020611

[4] Bekkhoucha SN, Cady A, Gautier P, Itim F, Donnio PY (2009) A portrait of Staphylococcus aureus from the other side of the Mediterranean Sea: molecular characteristics of isolates from Western Algeria. Eur J Clin Microbiol Infect Dis 28: 553–555. 19002727 10.1007/s10096-008-0660-x

[5] Udo EE, O’Brien FG, Al-Sweih N, Noronha B, Matthew B, et al (2008) Genetic lineages of community-associated methicillin-resistant Staphylococcus aureus in Kuwait hospitals. J Clin Microbiol 46: 3514–3516. 18632906 10.1128/JCM.00966-08 PMC2566081

[6] Boyle-Vavra S, Ereshefsky B, Wang CC, Daum RS (2005) Successful multiresistant community-associated methicillin-resistant Staphylococcus aureus lineage from Taipei, Taiwan, that carries either the novel Staphylococcal chromosome cassette mec (SCCmec) type VT or SCCmec type IV. J Clin Microbiol 43: 4719–4730. 16145133 10.1128/JCM.43.9.4719-4730.2005 PMC1234068

[7] Collignon P, Gosbell I, Vickery A, Nimmo G, Stylianopoulos T, et al (1998) Community-acquired meticillin-resistant Staphylococcus aureus in Australia. Australian Group on Antimicrobial Resistance. Lancet 352: 145–146. 9672301 10.1016/s0140-6736(98)85051-4

[8] Riley D, MacCulloch D, Morris AJ (1998) Methicillin-resistant S. aureus in the suburbs. N Z Med J 111: 59. 9539922

[9] Ellington MJ, Ganner M, Warner M, Cookson BD, Kearns AM (2010) Polyclonal multiply antibiotic-resistant methicillin-resistant Staphylococcus aureus with Panton-Valentine leucocidin in England. J Antimicrob Chemother 65: 46–50. 19887459 10.1093/jac/dkp386

[10] Nimmo GR, Coombs GW (2008) Community-associated methicillin-resistant Staphylococcus aureus (MRSA) in Australia. Int J Antimicrob Agents 31: 401–410. 18342492 10.1016/j.ijantimicag.2007.08.011

[11] Tristan A, Bes M, Meugnier H, Lina G, Bozdogan B, et al (2007) Global distribution of Panton-Valentine leukocidin–positive methicillin-resistant Staphylococcus aureus, 2006. Emerg Infect Dis 13: 594–600. 17553275 10.3201/eid1304.061316 PMC2725977

[12] Munckhof WJ, Schooneveldt J, Coombs GW, Hoare J, Nimmo GR (2003) Emergence of community-acquired methicillin-resistant Staphylococcus aureus (MRSA) infection in Queensland, Australia. Int J Infect Dis 7: 259–264. 14656416 10.1016/s1201-9712(03)90104-4

[13] Coombs GW, Nimmo GR, Pearson JC, Christiansen KJ, Bell JM, et al (2009) Prevalence of MRSA strains among Staphylococcus aureus isolated from outpatients, 2006. Commun Dis Intell 33: 10–20. 10.33321/cdi.2009.33.2 19618763

[14] Peleg AY, Munckhof WJ, Kleinschmidt SL, Stephens AJ, Huygens F (2005) Life-threatening community-acquired methicillin-resistant Staphylococcus aureus infection in Australia. Eur J Clin Microbiol Infect Dis 24: 384–387. 15926063 10.1007/s10096-005-1329-3

[15] Risson DC, O’Connor ED, Guard RW, Schooneveldt JM, Nimmo GR (2007) A fatal case of necrotising pneumonia due to community-associated methicillin-resistant Staphylococcus aureus. Med J Aust 186: 479–480. 17484712 10.5694/j.1326-5377.2007.tb01002.x

[16] Chua K, Seemann T, Harrison PF, Davies JK, Coutts SJ, et al (2010) Complete genome sequence of Staphylococcus aureus strain JKD6159, a unique Australian clone of ST93-IV community methicillin-resistant Staphylococcus aureus. J Bacteriol 192: 5556–5557. 20729356 10.1128/JB.00878-10 PMC2950503

[17] Chua KY, Seemann T, Harrison PF, Monagle S, Korman TM, et al (2011) The dominant Australian community-acquired methicillin-resistant Staphylococcus aureus clone ST93-IV [2B] is highly virulent and genetically distinct. PLoS One 6: e25887. 21991381 10.1371/journal.pone.0025887 PMC3185049

[18] Ellington MJ, Ganner M, Warner M, Boakes E, Cookson BD, et al (2010) First international spread and dissemination of the virulent Queensland community-associated methicillin-resistant Staphylococcus aureus strain. Clin Microbiol Infect 16: 1009–1012. 19624515 10.1111/j.1469-0691.2009.02994.x

[19] Coombs GW, Pearson JC, O’Brien FG, Murray RJ, Grubb WB, et al (2006) Methicillin-resistant Staphylococcus aureus clones, Western Australia. Emerg Infect Dis 12: 241–247. 16494749 10.3201/eid1202.050454 PMC3373111

[20] O’Brien FG, Coombs GW, Pearman JW, Gracey M, Moss F, et al (2009) Population dynamics of methicillin-susceptible and -resistant Staphylococcus aureus in remote communities. J Antimicrob Chemother 64: 684–693. 19713400 10.1093/jac/dkp285 PMC2740637

[21] Costa AM, Kay I, Palladino S (2005) Rapid detection of mecA and nuc genes in staphylococci by real-time multiplex polymerase chain reaction. Diagn Microbiol Infect Dis 51: 13–17. 15629224 10.1016/j.diagmicrobio.2004.08.014

[22] CLSI (2009) Performance standards for antimicrobial disk susceptibility tests. 7th ed Approved standard M02-A10. CLSI, Wayne, PA.

[23] CLSI (2009) Performance standards for antimicrobial susceptibility testing. 19th informational supplement M100-S18. CLSI, Wayne, PA.

[24] CA-SFM (1996) Report of the Comité de l’Antibiogramme de la Société Française de Microbiologie. Clin Microbiol Infect 2. 10.1111/j.1469-0691.1997.tb00639.x 11864145

[25] Finlay JE, Miller LA, Poupard JA (1997) Interpretive criteria for testing susceptibility of staphylococci to mupirocin. Antimicrob Agents Chemother 41: 1137–1139. 9145883 10.1128/aac.41.5.1137 PMC163864

[26] O’Brien FG, Udo EE, Grubb WB (2006) Contour-clamped homogeneous electric field electrophoresis of Staphylococcus aureus. Nat Protoc 1: 3028–3033. 17406564 10.1038/nprot.2006.382

[27] Enright MC, Day NP, Davies CE, Peacock SJ, Spratt BG (2000) Multilocus sequence typing for characterization of methicillin-resistant and methicillin-susceptible clones of Staphylococcus aureus. J Clin Microbiol 38: 1008–1015. 10698988 10.1128/jcm.38.3.1008-1015.2000 PMC86325

[28] Harmsen D, Claus H, Witte W, Rothganger J, Turnwald D, et al (2003) Typing of methicillin-resistant Staphylococcus aureus in a university hospital setting by using novel software for spa repeat determination and database management. J Clin Microbiol 41: 5442–5448. 14662923 10.1128/JCM.41.12.5442-5448.2003 PMC309029

[29] Benson G (1997) Sequence alignment with tandem duplication. Journal of computational biology : a journal of computational molecular cell biology 4: 351–367. 9278065 10.1089/cmb.1997.4.351

[30] Monecke S, Jatzwauk L, Weber S, Slickers P, Ehricht R (2008) DNA microarray-based genotyping of methicillin-resistant Staphylococcus aureus strains from Eastern Saxony. Clin Microbiol Infect 14: 534–545. 18373691 10.1111/j.1469-0691.2008.01986.x

[31] Monecke S, Slickers P, Ehricht R (2008) Assignment of Staphylococcus aureus isolates to clonal complexes based on microarray analysis and pattern recognition. FEMS Immunol Med Microbiol 53: 237–251. 18507678 10.1111/j.1574-695X.2008.00426.x

[32] Coombs GW, Monecke S, Ehricht R, Slickers P, Pearson JC, et al (2010) Differentiation of clonal complex 59 community-associated methicillin-resistant Staphylococcus aureus in Western Australia. Antimicrob Agents Chemother 54: 1914–1921. 20211891 10.1128/AAC.01287-09 PMC2863625

[33] Elements IWGotCoSCC (2009) Classification of staphylococcal cassette chromosome mec (SCCmec): guidelines for reporting novel SCCmec elements. Antimicrob Agents Chemother 53: 4961–4967. 19721075 10.1128/AAC.00579-09 PMC2786320

[34] Fey PD, Said-Salim B, Rupp ME, Hinrichs SH, Boxrud DJ, et al (2003) Comparative molecular analysis of community- or hospital-acquired methicillin-resistant Staphylococcus aureus. Antimicrob Agents Chemother 47: 196–203. 12499191 10.1128/AAC.47.1.196-203.2003 PMC149027

[35] Ma XX, Ito T, Kondo Y, Cho M, Yoshizawa Y, et al (2008) Two different Panton-Valentine leukocidin phage lineages predominate in Japan. J Clin Microbiol 46: 3246–3258. 18685010 10.1128/JCM.00136-08 PMC2566072

[36] Boakes E, Kearns AM, Ganner M, Perry C, Hill RL, et al (2011) Distinct bacteriophages encoding Panton-Valentine leukocidin (PVL) among international methicillin-resistant Staphylococcus aureus clones harboring PVL. J Clin Microbiol 49: 684–692. 21106787 10.1128/JCM.01917-10 PMC3043515

[37] Wolter DJ, Tenover FC, Goering RV (2007) Allelic variation in genes encoding Panton-Valentine leukocidin from community-associated Staphylococcus aureus. Clin Microbiol Infect 13: 827–830. 17610602 10.1111/j.1469-0691.2007.01763.x

[38] Goering RV, Morrison D, Al-Doori Z, Edwards GF, Gemmell CG (2008) Usefulness of mec-associated direct repeat unit (dru) typing in the epidemiological analysis of highly clonal methicillin-resistant Staphylococcus aureus in Scotland. Clin Microbiol Infect 14: 964–969. 18828855 10.1111/j.1469-0691.2008.02073.x

[39] Kennedy AD, Otto M, Braughton KR, Whitney AR, Chen L, et al (2008) Epidemic community-associated methicillin-resistant Staphylococcus aureus: recent clonal expansion and diversification. Proc Natl Acad Sci U S A 105: 1327–1332. 18216255 10.1073/pnas.0710217105 PMC2234137

[40] Coombs GW, Monecke S, Pearson JC, Tan HL, Chew YK, et al (2011) Evolution and diversity of community-associated methicillin-resistant Staphylococcus aureus in a geographical region. BMC Microbiol 11: 215. 21955438 10.1186/1471-2180-11-215 PMC3197503

[41] Munckhof WJ, Nimmo GR, Carney J, Schooneveldt JM, Huygens F, et al (2008) Methicillin-susceptible, non-multiresistant methicillin-resistant and multiresistant methicillin-resistant Staphylococcus aureus infections: a clinical, epidemiological and microbiological comparative study. Eur J Clin Microbiol Infect Dis 27: 355–364. 18278529 10.1007/s10096-007-0449-3

[42] Tong SY, Lilliebridge RA, Holt DC, McDonald MI, Currie BJ, et al (2009) High-resolution melting analysis of the spa locus reveals significant diversity within sequence type 93 methicillin-resistant Staphylococcus aureus from northern Australia. Clin Microbiol Infect 15: 1126–1131. 19392885 10.1111/j.1469-0691.2009.02732.x

[43] Tenover FC, Goering RV (2009) Methicillin-resistant Staphylococcus aureus strain USA300: origin and epidemiology. J Antimicrob Chemother 64: 441–446. 19608582 10.1093/jac/dkp241

[44] Tenover FC, Tickler IA, Goering RV, Kreiswirth BN, Mediavilla JR, et al (2012) Characterization of Nasal and Blood Culture Isolates of Methicillin-Resistant Staphylococcus aureus from Patients in United States Hospitals. Antimicrob Agents Chemother 56: 1324–1330. 22155818 10.1128/AAC.05804-11 PMC3294931

