# Supplementary material for: The Molecular Epidemiology of the Highly Virulent ST93 Australian Community Staphylococcus aureus Strain
Source: PLoS One. 2012 Aug 10;7(8):e43037. doi: 10.1371/journal.pone.0043037 (PMC3416834; doi:10.1371/journal.pone.0043037)
Supplement: Table S1 — Microarray DNA ST93 virulence profile (DOCX) [file pone.0043037.s001.docx]

| **Supplementary Table 1: ST93 virulence profile** | | | | | | | | | | | |
| --- | --- | --- | --- | --- | --- | --- | --- | --- | --- | --- | --- |
|  |  | **lukF/lukS PV** | **Superantigenic Toxins** | | **Leucocidins and Haemolysins** | **sak/chp/scn** | **et** | **edin** | **Proteases** | **Biofilms** | **ACME** |
| **Region** | **Reference**  **Number** |  | **Enterotoxins** | **tst** |  |  |  |  |  |  |  |
| **ST93 MSSA** | | | | | | | | | | | |
| NT | WBG7735 | *lukF/lukS PV* |  |  | *lukX/Y, hl, hla, hlb, hld, hlIII* | *sak, chp, scn* |  |  | *aur, splA, splE, sspA, sspB, sspP* | *icaA, icaC, icaD* |  |
| NT | WBG7762 | *lukF/lukS PV* |  |  | *lukX/(Y), hl,( hla), hlb, hld, hlIII* | *sak, chp, scn* |  |  | *aur, splA,( splE), sspA, sspB, sspP* | *icaA, icaC, icaD* |  |
| Qld | UQ40 | *lukF/lukS PV* |  |  | *lukX/Y, hl, hla, hlb, hld, hlIII* | *sak, chp, scn* |  |  | *aur, splA, splE, sspA, sspB, sspP* | *icaA, icaC, icaD* |  |
| Vic | DP2039 | *lukF/lukS PV* | entCM14 |  | *lukD, lukX/Y, hl, hla, hlb, hld, hlIII* | *sak, chp, scn* |  |  | *aur, splA, splE, sspA, sspB, sspP* | *icaA, icaC, icaD* |  |
| WA | C229T | *lukF/lukS PV* |  |  | *lukX, hl, (hla), hlb, hld, hlIII* | *sak, chp, scn* |  |  | *aur, splA, sspA, sspB, sspP* | *icaA, icaC, icaD* |  |
| WA | N126W | *lukF/lukS PV* |  |  | *lukX/Y,( hl),(hla), hlb, hld, hlIII* | *sak, chp, scn* |  |  | *aur, splA, splE, sspA, sspB, sspP* | *icaA, icaC, icaD* |  |
| WA | W17S | *lukF/lukS PV* |  |  | *lukX/Y, hl, hla, hlb, hld, hlIII* | *sak, chp, scn* |  |  | *aur, splA, splE, sspA, sspB, sspP* | *icaA, icaC, icaD* |  |
| WA | 113S | *lukF/lukS PV* |  |  | *(lukD), lukX/Y, hl, hla, hlb, hld, hlIII* | *sak, chp, scn* |  |  | *aur, splA, splE, sspA, sspB, sspP* | *icaA, icaC, icaD* |  |
| WA | 9506160A | *lukF/lukS PV* | entCM14 |  | *lukD, lukX/Y, hl, hla, hlb, hld, hlIII* | *sak, chp, scn* |  |  | *aur, splA, splE, sspA, sspB, sspP* | *icaA, icaC, icaD* |  |
| WA | 9509712N | *lukF/lukS PV* |  |  | *lukX/Y, hl, hla, hlb, hld, hlIII* | *sak, chp, scn* |  |  | *aur, splA, splE, sspA, sspB, sspP* | *icaA, icaC, icaD* |  |
| WA | 9524093R | *lukF/lukS PV* | (entCM14) |  | *(lukD), lukX/Y, hl, hla, hlb, hld, hlIII* | *sak, chp, scn* |  |  | *aur, splA, splE, sspA, sspB, sspP* | *icaA, icaC, icaD* |  |
| WA | 9525206A | *lukF/lukS PV* |  |  | *(lukD), lukX/Y, hl, hla, hlb, hld, hlIII* | *sak, chp, scn* |  |  | *aur, splA, splE, sspA, sspB, sspP* | *icaA, icaC, icaD* |  |
| WA | 9529120L | *lukF/lukS PV* | (entCM14) |  | *(lukD), lukX/Y, hl, hla, hlb, hld, hlIII* | *sak, chp, scn* |  |  | *aur, splA, splE, sspA, sspB, sspP* | *icaA, icaC, icaD* |  |
| **ST93 MRSA** | | | | | | | | | | | |
| ACT | SAPTCH92 | *lukF/lukS PV* | entCM14 |  | *lukD, lukX/Y, hl, hla, hlb, hld, hlIII* | *sak, chp, scn* |  |  | *aur, splA, splE, sspA, sspB, sspP* | *icaA, icaC, icaD* |  |
| ACT | SAPTCH53 | *lukF/lukS PV* | entCM14 |  | *lukD, lukX/Y, hl, hla, hlb, hld, hlIII* | *sak, chp, scn* |  |  | *aur, splA, splE, sspA, sspB, sspP* | *icaA, icaC, icaD* |  |
| NSW | SAPRPAH96 | *lukF/lukS PV* | (entCM14) |  | *lukD, lukX/Y, hl, hla, hlb, hld, hlIII* | *sak, chp, scn* |  |  | *aur, splA, splE, sspA, sspB, sspP* | *icaA, icaC, icaD* |  |
| NSW | SAPWH23 | *lukF/lukS PV* |  |  | *lukD, lukX, hl, hla, hlb, hld, hlIII* | *sak, chp, scn* |  |  | *aur, splA, splE, sspA, sspB, sspP* | *icaA, icaC, icaD* |  |
| NSW | SAPWH39 | *lukF/lukS PV* | entCM14 |  | *(lukD), lukX/Y, hl, hla, hlb, hld, hlIII* | *sak, chp, scn* |  |  | *aur, splA, splE, sspA, sspB, sspP* | *icaA, icaC, icaD* |  |
| NSW | SAPWH61 | *lukF/lukS PV* | entCM14 |  | *lukD, lukX, hl, hla, hlb, hld, hlIII* | *sak, chp, scn* |  |  | *aur, splA, sspA, sspB, sspP* | *icaA, icaC, icaD* |  |
| NSW | SAPWH64 | *lukF/lukS PV* | (entCM14) |  | *lukD, lukX/Y, hl, hla, hlb, hld, hlIII* | *sak, chp, scn* |  |  | *aur, splA, splE, sspA, sspB, sspP* | *icaA, icaC, icaD* |  |
| NSW | SAPWH94 | *lukF/lukS PV* | entCM14 |  | *lukD, lukX/Y, hl, hla, hlb, hld, hlIII* | *sak, chp, scn* |  |  | *aur, splA, splE, sspA, sspB, sspP* | *icaA, icaC, icaD* |  |
| NSW | SAPWH71 |  |  |  | *lukX, hl, hla, hlb, hld, hlIII* | *sak, chp, scn* |  |  | *aur, splA, (splE), sspA, sspB, sspP* | *icaA, icaC, icaD* |  |
| NSW | SAPCRGH95 | *lukF/lukS PV* |  |  | *hlb, hld, hlIII* | *sak, chp, scn* |  |  | *aur, splA, sspA, sspB, sspP* | *icaA, icaC, icaD* |  |
| NSW | SAPRPAH21 | *lukF/lukS PV* | (entCM14) |  | *(lukD), lukX/Y, hl, hla, hlb, hld, hlIII* | *sak, chp, scn* |  |  | *aur, splA, splE, sspA, sspB, sspP* | *icaA, icaC, icaD* |  |
| NSW | SAPRPAH7 | *lukF/lukS PV* | entCM14 |  | *lukD, lukX/Y, hl, hla, hlb, hld, hlIII* | *sak, chp, scn* |  |  | *aur, splA, splE, sspA, sspB, sspP* | *icaA, icaC, icaD* |  |
| NSW | SAPWH10 | *lukF/lukS PV* | entCM14 |  | *lukD, lukX/Y, hl, hla, hlb, hld, hlIII* | *sak, chp, scn* |  |  | *aur, splA, splE, sspA, sspB, sspP* | *icaA, icaC, icaD* |  |
| NSW | SAPWH53 |  |  |  | *lukX/Y, hl, hla, hlb, hld, hlIII* | *sak, chp, scn* |  |  | *aur, splA, splE, sspA, sspB, sspP* | *icaA, icaC, icaD* |  |
| NT | SAPRDH61 | *lukF/lukS PV* |  |  | *lukX/(Y), hl, hlb, hld, hlIII* | *sak, chp, scn* |  |  | *aur, splA, (splE), sspA, sspB, sspP* | *icaA, icaC, icaD* |  |
| NT | SAPRDH27 | *lukF/lukS PV* |  |  | *lukX/Y, hl,( hla), hlb, hld, hlIII* | *sak, chp, scn* |  |  | *aur, splA, splE, sspA, sspB, sspP* | *icaA, icaC, icaD* |  |
| NT | SAPRDH2 | *lukF/lukS PV* | entCM14 |  | *lukD, lukX/Y, hl, hla, hlb, hld, hlIII* | *sak, chp, scn* |  |  | *aur, splA, splE, sspA, sspB, sspP* | *icaA, icaC, icaD* |  |
| Qld | SAPRBH98 | *lukF/lukS PV* | entCM14 |  | *lukD, lukX/Y, hl, hla, hlb, hld, hlIII* | *sak, chp, scn* |  |  | *aur, splA, splE, sspA, sspB, sspP* | *icaA, icaC, icaD* |  |
| Qld | SAPRBH12 | *lukF/lukS PV* |  |  | *lukX, hl, hlb, hld, hlIII* | *sak, chp, scn* |  |  | *aur, splA, sspA, sspB, sspP* | *icaA, icaC, icaD* |  |
| Qld | SAPGCH3 | *lukF/lukS PV* |  |  | *lukX/(Y),( hl), hla, hlb, hld, hlIII* | *sak, chp, scn* |  |  | *aur, splA,( splE), sspA, sspB, sspP* | *icaA, icaC, icaD* |  |
| Qld | SAPRBH14 | *lukF/lukS PV* |  |  | *lukX, hl, hlb, hld, hlIII* | *sak, chp, scn* |  |  | *aur, splA, splE, sspA, sspB, sspP* | *icaA, icaC, icaD* |  |
| Qld | SAPCBH10 | *lukF/lukS PV* | entCM14 |  | *lukD, lukX/Y, hl, hla, hlb, hld, hlIII* | *sak, chp, scn* |  |  | *aur, splA, splE, sspA, sspB, sspP* | *icaA, icaC, icaD* |  |
| Qld | SAPGCH28 | *lukF/lukS PV* | (entCM14) |  | *lukD, lukX/Y, hl, hla, hlb, hld, hlIII* | *sak, chp, scn* |  |  | *aur, splA, splE, sspA, sspB, sspP* | *icaA, icaC, icaD* |  |
| Qld | SAPRBH1 | *lukF/lukS PV* | (entCM14) |  | *lukD, lukX/Y, hl, hla, hlb, hld, hlIII* | *sak, chp, scn* |  |  | *aur, splA, splE, sspA, sspB, sspP* | *icaA, icaC, icaD* |  |
| SA | SAPGPSA73 | *lukF/lukS PV* | (entCM14) |  | *lukD, lukX/Y, hl, hla, hlb, hld, hlIII* | *sak, chp, scn* |  |  | *aur, splA, splE, sspA, sspB, sspP* | *icaA, icaC, icaD* |  |
| SA | SAPIMVS24 | *lukF/lukS PV* | (entCM14) |  | *lukX/Y, hl, hla, hlb, hld, hlIII* | *sak, chp, scn* |  |  | *aur, splA, splE, sspA, sspB, sspP* | *icaA, icaC, icaD* |  |
| SA | SAPIMVS31 | *lukF/lukS PV* | (entCM14) |  | *lukD, lukX/Y, hl, hla, hlb, hld, hlIII* | *sak, chp, scn* |  |  | *aur, splA, splE, sspA, sspB, sspP* | *icaA, icaC, icaD* |  |
| Vic | SAPRCH74 | *lukF/lukS PV* | entCM14 |  | *lukD, lukX/Y, hl, hla, hlb, hld, hlIII* | *sak, chp, scn* |  |  | *aur, splA, splE, sspA, sspB, sspP* | *icaA, icaC, icaD* |  |
| Vic | SAPAH21 | *lukF/lukS PV* |  |  | *(lukD), lukX/Y, hl, hla, hlb, hld, hlIII* | *sak, chp, scn* |  |  | *aur, splA, splE, sspA, sspB, sspP* | *icaA, icaC, icaD* |  |
| WA | 16790 | *lukF/lukS PV* | entCM14 |  | *lukD, lukX/Y, hl, hla, hlb, hld, hlIII* | *sak, chp, scn* |  |  | *aur, splA, splE, sspA, sspB, sspP* | *icaA, icaC, icaD* |  |
| WA | 16815 | *lukF/lukS PV* | entCM14 |  | *lukD/(E), lukX/Y, hl, hla, hlb, hld, hlIII untruncated hlb* | *sak, chp, scn* |  |  | *aur, splA, splE, sspA, sspB, sspP* | *icaA, icaC, icaD* |  |
| WA | 15586 | *lukF/lukS PV* |  |  | *lukX/(Y), hl,( hla), hlb, hld, hlIII* | *sak, chp, scn* |  |  | *aur, splA, sspA, sspB, sspP* | *icaA, icaC, icaD* |  |
| WA | 15587 | *lukF/lukS PV* |  |  | *(lukD), lukX/(Y), hl, hla, hlb, hld, hlIII* | *sak, chp, scn* |  |  | *aur, splA, splE, sspA, sspB, sspP* | *icaA, icaC, icaD* |  |
| WA | 16414 | *lukF/lukS PV* |  |  | *lukX/Y, hl, hla, hlb, hld, hlIII* | *sak, chp, scn* |  |  | *aur, splA, splE, sspA, sspB, sspP* | *icaA, icaC, icaD* |  |
| WA | 16475 | *lukF/lukS PV* |  |  | *(lukD), lukX/Y, hl, hla, hlb, hld, hlIII* | *sak, chp, scn* |  |  | *aur, splA, splE, sspA, sspB, sspP* | *icaA, icaC, icaD* |  |
| WA | 17164 | *lukF/lukS PV* | entCM14 |  | *lukD/(E), lukX/Y, hl, hla, hlb, hld, hlII, untuncated hlbI* | *sak, chp, scn* |  |  | *aur, splA, splE, sspA, sspB, sspP* | *icaA, icaC, icaD* |  |
| WA | 18158 | *lukF/lukS PV* | entCM14 |  | *lukD, lukX/Y, hl, hla, hlb, hld, hlIII* | *sak, chp, scn* |  |  | *aur, splA, splE, sspA, sspB, sspP* | *icaA, icaC, icaD* |  |
| WA | 18385 | *lukF/lukS PV* |  |  | *lukX/(Y), hl,( hla), hlb, hld, hlIII* | *sak, chp, scn* |  |  | *aur, splA,( splE), sspA, sspB, sspP* | *icaA, icaC, icaD* |  |
| WA | 18418 | *lukF/lukS PV* |  |  | *lukD, lukX/Y, hl, (hla), hlb, hld, hlIII* | *sak, chp, scn* |  |  | *aur, splA, splE, sspA, sspB, sspP* | *icaA, icaC, icaD* |  |
| WA | 20198 |  | entCM14 |  | *lukD, lukX/Y, hl, hla, hlb, hlIII* | *sak, chp, scn* |  |  | *aur, splA, splE, sspA, sspB, sspP* | *icaA, icaC, icaD* |  |
| WA | SAPRPH48 | *lukF/lukS PV* | (entCM14) |  | *lukD, lukX/Y, hl, hla, hlb, hld, hlIII* | *sak, chp, scn* |  |  | *aur, splA, splE, sspA, sspB, sspP* | *icaA, icaC, icaD* |  |
| WA | 16908 | *lukF/lukS PV* | entCM14 |  | *(hlgA)lukD/E, lukX/Y, hl, hla, hlb, hld, hlIII, untruncated hlb* | *sak, chp, scn* |  |  | *aur, splA, splE, sspA, sspB, sspP* | *icaA, icaC, icaD* |  |
| WA | 17090 | *lukF/lukS PV* | entCM14 |  | *lukD, lukX/Y, hl, hla, hlb, hld, hlIII* | *sak, chp, scn* |  |  | *aur, splA, splE, sspA, sspB, sspP* | *icaA, icaC, icaD* |  |
| WA | 17195 | *lukF/lukS PV* | entCM14 |  | *(lukD), lukX/Y, hl, hla, hlb, hld, hlIII* | *sak, chp, scn* |  |  | *aur, splA, splE, sspA, sspB, sspP* | *icaA, icaC, icaD* |  |
| WA | 20548 | *lukF/lukS PV* | (entCM14) |  | *lukX/Y, hl, hla, hlb, hld, hlIII* | *sak, chp, scn* |  |  | *aur, splA, splE, sspA, sspB, sspP* | *icaA, icaC, icaD* |  |
| **Control Strain** | | | | | | | | | | | |
| Vic | JKD6159 | *lukF/lukS PV* | entCM14 |  | *(lukD), lukX/Y, hl, hla, hlb, hld, hlIII* | *sak, chp, scn* |  |  | *aur, splA, splE, sspA, sspB, sspP* | *icaA, icaC, icaD* |  |

Regions: ACT, Australian Capital Territory; NSW, New South Wales; NT, Northern Territory, Qld, Queensland; SA, South Australia; Vic, Victoria; WA, Western Australia

*lukF*/*lukS* PV, Panton Valentine leucocidin F and S component genes; entCM14, enterotoxin-like protein ORF CM14; *lukD/E*, leucocidin D and E component genes; *lukX/ Y*, leucocidin haemolysin toxin family protein genes; *hl*, putative membrane protein gene; *hla*, haemolysin alpha gene, *hlb*, haemolysin beta gene; *hld*, haemolysin delta gene; *hlIII*, putative membrane protein; sak, staphylokinase gene; chp, chemotaxis-inhibiting protein (CHIPS) gene; *scn*, staphylococcal complement inhibitor gene; *aur* aureolysin gene; *splA*, serinprotease A gene; *splE*, serinprotease E gene, *sspA*, glutamylendopeptidase gene, *sspB*, staphopain B protease gene, *sspP*, staphopain A protease gene, *icaA*, intracellular adhesion protein A gene; *icaC*, intracellular adhesion protein C gene; *icaD*, biofilm PIA synthesis protein D gene

( ), gene detected but yielding weak or ambiguous signals
