# Supplementary material for: The Molecular Epidemiology of the Highly Virulent ST93 Australian Community Staphylococcus aureus Strain
Source: PLoS One. 2012 Aug 10;7(8):e43037. doi: 10.1371/journal.pone.0043037 (PMC3416834; doi:10.1371/journal.pone.0043037)
Supplement: Table S2 — Microarray DNA ST93 ST93 staphylococcal superantigen/enterotoxin-like genes (set/ssl) profile. (DOCX) [file pone.0043037.s002.docx]

| **Supplementary Table 2: ST93 staphylococcal superantigen/enterotoxin-like genes (set/ssl) profile** | | | | | | | | | | | | | | | | | | | | | | | | | | | | | | | | | | | | |
| --- | --- | --- | --- | --- | --- | --- | --- | --- | --- | --- | --- | --- | --- | --- | --- | --- | --- | --- | --- | --- | --- | --- | --- | --- | --- | --- | --- | --- | --- | --- | --- | --- | --- | --- | --- | --- |
| **Region** | **Reference**  **Number** | **setC (MW0345)** | **ssl01/set6 (COL/SACOL468)** | **ssl01/set6 (Mu50/SAV0422)** | **ssl01/set6 (MW2-MW0382)** | **ssl01/set6 (SAR0422/MRSA252)** | **ssl01/set6 (all others)** | **ssl01/set6 (RF122)** | **ssl02/set7** | **ssl03/set8** | **ssl03/set8 (MRSA252/SAR0424)** | **ssl04/set9** | **ssl04/set9 (MRSA252/SAR0425)** | **ssl05/set3** | **sl05/set3 (RF122)** | **ssl05/set3 (MRSA252)** | **ssl06/set21** | **ssl06/set21 (NCTC/MW2)** | **ssl07/set1** | **ssl07/set1 (MRSA252)** | **ssl07/set1 (AF188836)** | **ssl08/set12** | **ssl09/set5** | **ssl09/set5 (MRSA252)** | **ssl10/set4** | **ssl10/set4 (RF122)** | **Ssl10/set4 (MRSA252)** | **ssl11/set2 (COL)** | **ssl11/set2 (Mu50/N315)** | **ssl11/set2 (MW2/MSSA476)** | **ssl11/set2 (MRSA252)** | **setB3** | **setB3 (MRSA252)** | **setB2** | **setB2 (MRSA252)** | **setB1** |
| **ST93 MSSA** | | | | | | | | | | | | | | | | | | | | | | | | | | | | | | | | | | | | |
| NT | WBG7735 | + |  |  |  |  | w | + |  |  |  |  | w |  |  |  | + |  |  |  |  |  |  |  |  |  |  |  |  |  |  |  | + |  |  |  |
| NT | WBG7762 | + |  |  |  |  |  | w |  |  |  |  |  |  |  |  | + |  |  |  |  |  |  |  |  |  |  |  |  |  |  |  | w |  |  |  |
| Qld | UQ40 | + |  |  | + |  | + | + |  |  |  |  | w |  | w |  | + |  |  |  |  | w |  |  |  |  |  |  |  |  |  |  | + |  |  |  |
| Vic | DP32039 | + |  |  | + |  | + | + |  |  |  |  | + |  | + |  | + | + |  |  |  | + |  |  | + |  | + |  |  |  |  |  | + |  |  | w |
| WA | C229T | + |  |  |  |  | w | + |  |  |  |  |  |  |  |  | + |  |  |  |  |  |  |  | + |  | + |  |  |  |  |  | + |  |  |  |
| WA | N126W | + |  |  |  |  | w | + |  |  |  |  |  |  |  |  | + |  |  |  |  |  |  |  |  |  |  |  |  |  |  |  | + |  |  |  |
| WA | W17S | + |  |  |  |  | + | + |  |  |  |  | + |  | w |  | + | w |  |  |  |  |  |  | + |  |  |  |  |  |  |  | + |  |  |  |
| WA | 113S | + |  |  | + |  | + | + |  |  |  |  | w |  | w |  | + |  |  |  |  |  |  |  | + |  |  |  |  |  |  |  | + |  |  |  |
| WA | 9506160A | + |  |  | + |  | + | + |  |  |  |  | + |  | w |  | + | + |  |  |  | + |  |  |  |  | + |  |  |  |  |  | + |  |  |  |
| WA | 9509712N | + |  |  | + |  | + | + |  |  |  |  | + |  |  |  | + | w |  |  |  | w |  |  | + |  |  |  |  |  |  |  | + |  |  |  |
| WA | 9524093R | + |  |  | + |  | + | + |  |  |  |  | + |  |  |  | + | + |  |  |  | w |  |  | + |  |  |  |  |  |  |  | + |  |  |  |
| WA | 9525206A | + |  |  | + |  | + | + |  |  |  |  | w |  |  |  | + |  |  |  |  |  |  |  |  |  |  |  |  |  |  |  | + |  |  |  |
| WA | 9529120L | + |  |  | + |  | + | + |  |  |  |  | + |  |  |  | + | w |  |  |  | w |  |  | + |  | + |  |  |  |  |  | + |  |  |  |
| **ST 93 MRSA** | | | | | | | | | | | | | | | | | | | | | | | | | | | | | | | | | | | | |
| ACT | SAPTCH92 | + |  |  | + |  | + | + |  |  |  |  | + |  | w |  | + | + |  |  |  | + |  |  | + |  | + |  |  |  |  |  | + |  |  |  |
| ACT | SAPTCH53 | + |  |  | + |  | + | + |  |  |  |  | + |  | w |  | + | + |  |  |  | + |  |  |  |  | + |  |  |  |  |  | + |  |  |  |
| NSW | SAPRPAH96 | + |  |  | + |  | + | + |  |  |  |  | w |  |  |  | + | w |  |  |  |  |  |  |  |  |  |  |  |  |  |  | + |  |  |  |
| NSW | SAPWH23 | + |  |  |  |  |  | + |  |  |  |  |  |  |  |  | + |  |  |  |  |  |  |  |  |  |  |  |  |  |  |  |  |  |  |  |
| NSW | SAPWH39 | + |  |  | + |  | + | + |  |  |  |  | + |  |  |  | + | + |  |  |  | + |  |  | + |  | + |  |  |  |  |  | + |  |  |  |
| NSW | SAPWH61 | + |  |  |  |  |  | + |  |  |  |  |  |  |  |  | + |  |  |  |  |  |  |  | + |  |  |  |  |  |  |  | + |  |  |  |
| NSW | SAPWH64 | + |  |  | + |  | + | + |  |  |  |  | + |  |  |  | + | w |  |  |  | + |  |  |  |  |  |  |  |  |  |  | + |  |  |  |
| NSW | SAPWH94 | + |  |  | + |  | + | + |  |  |  |  | + |  | w |  | + | + |  |  |  | + |  |  | + |  | + |  |  |  |  |  | + |  |  |  |
| NSW | SAPWH71 | + |  |  |  |  |  | + |  |  |  |  |  |  |  |  | + |  |  |  |  |  |  |  |  |  |  |  |  |  |  |  |  |  |  |  |
| NSW | SAPCRGH95 | + |  |  |  |  |  |  |  |  |  |  |  |  |  |  | + |  |  |  |  |  |  |  |  |  |  |  |  |  |  |  |  |  |  |  |
| NSW | SAPRPAH21 | + |  |  | + |  | + | + |  |  |  |  | + |  |  |  | + | w |  |  |  | w |  |  | + |  |  |  |  |  |  |  | + |  |  |  |
| NSW | SAPRPAH7 | + |  |  | + |  | + | + |  |  |  |  | + |  | w |  | + | + |  |  |  | + |  |  | + |  | + |  |  |  |  |  | + |  |  |  |
| NSW | SAPWH10 | + |  |  | + |  | + | + |  |  |  |  | + |  | w |  | + | + |  |  |  | + |  |  | + |  | + |  |  |  |  |  | + |  |  |  |
| NSW | SAPWH53 | + |  |  |  |  | w | + |  |  |  |  | w |  |  |  | + |  |  |  |  |  |  |  |  |  |  |  |  |  |  |  | + |  |  |  |
| NT | SAPRDH61 | + |  |  |  |  |  |  |  |  |  |  |  |  |  |  | + |  |  |  |  |  |  |  |  |  |  |  |  |  |  |  |  |  |  |  |
| NT | SAPRDH27 | + |  |  |  |  | w | w |  |  |  |  |  |  |  |  | + |  |  |  |  |  |  |  |  |  |  |  |  |  |  |  | w |  |  |  |
| NT | SAPRDH2 | + |  |  | + |  | + | + |  |  |  |  | + |  |  |  | + | w |  |  |  | + |  |  | + |  | + |  |  |  |  |  | + |  |  |  |
| Qld | SAPRBH98 | + |  |  | + |  | + | + |  |  |  |  | + |  | w |  | + | + |  |  |  | + |  |  | + |  | + |  |  |  |  |  | + |  |  |  |
| Qld | SAPRBH12 | + |  |  |  |  | + |  |  |  |  |  |  |  |  |  | + |  |  |  |  |  |  |  |  |  |  |  |  |  |  |  |  |  |  |  |
| Qld | SAPGCH3 | + |  |  |  |  |  | + |  |  |  |  |  |  |  |  | + |  |  |  |  |  |  |  |  |  |  |  |  |  |  |  |  |  |  |  |
| Qld | SAPRBH14 | + |  |  |  |  |  | w |  |  |  |  |  |  |  |  | + |  |  |  |  |  |  |  |  |  |  |  |  |  |  |  |  |  |  |  |
| Qld | SAPCBH10 | + |  |  | + |  | + | + |  |  |  |  | + |  | w |  | + | + |  |  |  | + |  |  |  |  | + |  |  |  |  |  | + |  |  |  |
| Qld | SAPGCH28 | + |  |  | + |  | + | + |  |  |  |  | + |  |  |  | + | w |  |  |  | + |  |  | + |  | + |  |  |  |  |  | + |  |  |  |
| Qld | SAPRBH1 | + |  |  | + |  | + | + |  |  |  |  | + |  |  |  | + | w |  |  |  | + |  |  | + |  |  |  |  |  |  |  | + |  |  |  |
| SA | GPSA73 | + |  |  | + |  | + | + |  |  |  |  | + |  |  |  | + | + |  |  |  | w |  |  | + |  |  |  |  |  |  |  | + |  |  |  |
| SA | SAPIMVS24 | + |  |  |  |  | w | + |  |  |  |  | + |  |  |  | + | w |  |  |  |  |  |  |  |  |  |  |  |  |  |  | + |  |  |  |
| SA | SAPIMVS31 | + |  |  | + |  | + | + |  |  |  |  | + |  |  |  | + | w |  |  |  | + |  |  | + |  | + |  |  |  |  |  | + |  |  |  |
| Vic | SAPRCH74 | + |  |  | + |  | + | + |  |  |  |  | + |  | w |  | + | + |  |  |  | + |  |  | + |  | + |  |  |  |  |  | + |  |  |  |
| Vic | SAPAH21 | + |  |  | + |  | + | + |  |  |  |  | + |  |  |  | + | w |  |  |  | + |  |  |  |  |  |  |  |  |  |  | + |  |  |  |
| WA | 16790 | + |  |  | + |  | + | + |  |  |  |  | + |  | w |  | + | + |  |  |  | + |  |  | + |  | + |  |  |  |  |  | + |  |  |  |
| WA | 16815 | + |  |  | + |  | + | + |  |  |  |  | + | w | + |  | + | + |  |  |  | + | w |  | + |  | + |  |  |  |  |  | + |  | w | + |
| WA | 15586 | + |  |  |  |  |  | w |  |  |  |  |  |  |  |  | + |  |  |  |  |  |  |  |  |  |  |  |  |  |  |  | w |  |  |  |
| WA | 15587 | + |  |  |  |  | w | + |  |  |  |  |  |  | w |  | + |  |  |  |  |  |  |  | + |  |  |  |  |  |  |  | w |  |  |  |
| WA | 16414 | + |  |  |  |  |  | + |  |  |  |  | w |  |  |  | + |  |  |  |  |  |  |  |  |  |  |  |  |  |  |  | + |  |  |  |
| WA | 16475 | + |  |  | + |  | + | + |  |  |  |  | + |  | + |  | + | w |  |  |  | w |  |  | + |  | + |  |  |  |  |  | + |  |  | w |
| WA | 17164 | + |  |  | + |  | + | + |  |  |  | w | + | w | + |  | + | + |  |  |  | + | + |  | + |  | + |  |  |  |  |  | + |  | + | + |
| WA | 18158 | + |  |  | + |  | + | + |  |  |  |  | + |  | w |  | + | + |  |  |  | + |  |  | + |  |  |  |  |  |  |  | + |  |  |  |
| WA | 18385 | + |  |  |  |  |  | w |  |  |  |  |  |  |  |  | + |  |  |  |  |  |  |  |  |  |  |  |  |  |  |  |  |  |  |  |
| WA | 18418 | + |  |  | + |  | + | + |  |  |  |  | + |  | + |  | + | + |  |  |  | + |  |  | + |  | + |  |  |  |  |  | + |  |  |  |
| WA | 20198 | + |  |  | + |  | + | + |  |  |  |  | + |  |  |  | + | + |  |  |  | + |  |  |  |  | + |  |  |  |  |  | + |  |  |  |
| WA | SAPRPH48 | + |  |  | + |  | + | + |  |  |  |  | + |  | + |  | + | + |  |  |  | + |  |  | + |  | + |  |  |  |  |  | + |  | w | w |
| WA | 16908 | + |  |  | + |  | + | + | w |  |  | + | + | w | + |  | + | + |  |  |  | + | + |  | + |  | + |  |  |  |  |  | + |  | + | + |
| WA | 17090 | + |  |  | + |  | + | + |  |  |  |  | + |  | w |  | + | + |  |  |  | + |  |  | + |  | + |  |  |  |  |  | + |  |  |  |
| WA | 17195 | + |  |  | + |  | + | + |  |  |  |  | + |  | w |  | + | + |  |  |  | + |  |  | + |  | + |  |  |  |  |  | + |  |  |  |
| WA | 20548 | + |  |  | + |  | + | + |  |  |  |  | + |  |  |  | + | w |  |  |  | w |  |  | + |  |  |  |  |  |  |  | + |  |  |  |
| **Control Strain** | | | | | | | | | | | | | | | | | | | | | | | | | | | | | | | | | | | | |
| Vic | JKD6159 | + |  |  | + |  | + | + |  |  |  |  | + |  |  |  | + | + |  |  |  | + |  |  |  |  |  |  |  |  |  |  | + |  |  |  |

Regions: ACT, Australian Capital Territory; NSW, New South Wales; NT, Northern Territory, Qld, Queensland; SA, South Australia; Vic, Victoria; WA, Western Australia

*setC*, staphylococcal exotoxin-like protein gene; *ssl01/set6*, staphylococcal superantigen-like protein 1 gene (alleles); *ssl021/set7*, staphylococcal superantigen-like protein 2 gene; *ssl03/set8*, staphylococcal superantigen-like protein 3 gene; *ssl04/set9*, staphylococcal superantigen-like protein 4 gene (alleles); *ssl51/set3*, staphylococcal superantigen-like protein 3 gene (alleles); *ssl06/set21*, staphylococcal superantigen-like protein 6 gene (alleles);

*ssl07/set1*, staphylococcal superantigen-like protein 7 gene (alleles); *ssl08/set6*, staphylococcal superantigen-like protein 8 gene; *ssl09/set9*, staphylococcal superantigen-like protein 9 gene (alleles); *ssl10/set4*, staphylococcal superantigen-like protein 10 gene (alleles); *ssl11/set2*, staphylococcal superantigen-like protein 1 gene (alleles); *setB3,* staphylococcal exotoxin-like protein gene, second locus (alleles); *setB2,* staphylococcal exotoxin-like protein gene, second locus (alleles); *setB1,* staphylococcal exotoxin-like protein gene

+, gene detected; w, gene detected but yielding weak or ambiguous signals
