# Supplementary material for: The Molecular Epidemiology of the Highly Virulent ST93 Australian Community Staphylococcus aureus Strain
Source: PLoS One. 2012 Aug 10;7(8):e43037. doi: 10.1371/journal.pone.0043037 (PMC3416834; doi:10.1371/journal.pone.0043037)
Supplement: Table S3 and S4 — Microarray DNA ST93 ST93 MSCRAMMs and adhesion profile. (DOCX) [file pone.0043037.s003.docx]

| **Supplementary Table 3: Microarray DNA ST93 MSCRAMMs and adhesion profile** | | | | | | | | | | | | | | | | | | | | | | | | | | | | | | | | | | | | |
| --- | --- | --- | --- | --- | --- | --- | --- | --- | --- | --- | --- | --- | --- | --- | --- | --- | --- | --- | --- | --- | --- | --- | --- | --- | --- | --- | --- | --- | --- | --- | --- | --- | --- | --- | --- | --- |
| **Region** | **Reference**  **Number** | **bbp (all)** | **bbp (COL /MW2)** | **bbp (MRSA252)** | **bbp (Mu50)** | **bbp (RF122)** | **bbp (ST45)** | **clfA (all)** | **clfA (COL/ RF122)** | **clfA (MRSA252)** | **clfaA (Mu50/MW2)** | **clfB- all)** | **clfB (COL + Mu50)** | **clfB (MW2)** | **clfB (RF122)** | **cna** | **ebh (all)** | **eno** | **fib** | **fib (MRSA252)** | **ebpS** | **epbS (01-1111)** | **ebpS (COL)** | **fnbA (a(l)** | **fnbA (COL)** | **fnbA (MRSA252)** | **fnbA (Mu50/MW2)** | **fnbA (RF122)** | **fnbB (COL)** | **fnbB (COL/Mu50/MW2)** | **fnbB (Mu50)** | **fnbB (MW2)** | **fnbB (ST15)** | **fnbB (ST45-2)** |  |  |
| **ST93 MSSA** | | | | | | | | | | | | | | | | | | | | | | | | | | | | | | | | | | | | |
| NT | WBG7735 | _+_ |  |  | _+_ |  |  | _+_ |  |  | + | + |  |  | + |  |  | + |  | + | + |  |  | + |  |  |  |  |  | + |  |  |  |  |  |  |
| NT | WBG7762 | _+_ |  |  | _+_ |  |  | _+_ |  |  | + | + |  |  | + |  |  | + |  | + | + |  |  | + |  |  |  |  |  | + |  |  |  |  |  |  |
| Qld | UQ40 | _+_ |  |  | _+_ |  |  | _+_ |  |  | + | + |  |  | + |  |  | + |  | + | + |  |  | + |  |  |  |  |  | + | w |  |  |  |  |  |
| Vic | DP2039 | _+_ |  |  | _+_ |  | w | _+_ |  |  | + | + |  |  | + |  |  | + |  | + | + |  | + | + |  |  |  |  |  | + | + |  |  |  |  |  |
| WA | C229T | _+_ |  |  | _+_ |  |  | _+_ |  |  | + | + |  |  | + |  |  | + |  | + | + |  |  | + |  |  |  |  |  | + |  |  |  |  |  |  |
| WA | N126W | _+_ |  |  | _+_ |  |  | _+_ |  |  | + | + |  |  | + |  |  | + |  | + | + |  |  | + |  |  |  |  |  | + |  |  |  |  |  |  |
| WA | W17S | _+_ |  |  | _+_ |  |  | _+_ |  |  | + | + |  |  | + |  |  | + |  | + | + |  |  | + |  |  |  |  |  | + |  |  |  |  |  |  |
| WA | Y113S | _+_ |  |  | _+_ |  |  | _+_ |  |  | + | + |  |  | + |  |  | + |  | + | + |  |  | + |  |  |  |  |  | + |  |  |  |  |  |  |
| WA | 9506160A | _+_ |  |  | _+_ |  |  | _+_ |  |  | + | + |  |  | + |  |  | + |  | + | + |  | + | + |  |  |  |  |  | + | + |  |  |  |  |  |
| WA | 9509712N | _+_ |  |  | _+_ |  |  | _+_ |  |  | + | + |  |  | + |  |  | + |  | + | + |  |  | + |  |  |  |  |  | + | w |  |  |  |  |  |
| WA | 9524093R | _+_ |  |  | _+_ |  |  | _+_ |  |  | + | + |  |  | + |  |  | + |  | + | + |  |  | + |  |  |  |  |  | + | w |  |  |  |  |  |
| WA | 9525206A | _+_ |  |  | _+_ |  |  | _+_ |  |  | + | + |  |  | + |  |  | + |  | + | + |  |  | + |  |  |  |  |  | + |  |  |  |  |  |  |
| WA | 9529120L | _+_ |  |  | _+_ |  |  | _+_ |  |  | + | + |  |  | + |  |  | + |  | + | + |  |  | + |  |  |  |  |  | + | w |  |  |  |  |  |
| **ST93 MRSA** | | | | | | | | | | | | | | | | | | | | | | | | | | | | | | | | | | | | |
| ACT | SAPTCH92 | _+_ |  |  | _+_ |  |  | _+_ |  |  | + | + |  |  | + |  |  | + |  | + | + |  | + | + |  |  |  |  |  | + | + |  |  |  |  |  |
| ACT | SAPTCH53 | _+_ |  |  | _+_ |  |  | _+_ |  |  | + | + |  |  | + |  |  | + |  | + | + |  | w | + |  |  |  |  |  | + | + |  |  |  |  |  |
| NSW | SAPRPAH96 | _+_ |  |  | _+_ |  |  | _+_ |  |  | + | + |  |  | + |  |  | + |  | + | + |  |  | + |  |  |  |  |  | + |  |  |  |  |  |  |
| NSW | SAPWH23 | _+_ |  |  | _+_ |  |  | _+_ |  |  | + | + |  |  | + |  |  | + |  | + | + |  |  | + |  |  |  |  |  | + |  |  |  |  |  |  |
| NSW | SAPWH39 | _+_ |  |  | _+_ |  |  | _+_ |  |  | + | + |  |  | + |  |  | + |  | + | + |  | + | + |  |  |  |  |  | + | + |  |  |  |  |  |
| NSW | SAPWH61 | _+_ |  |  | _+_ |  |  | _+_ |  |  | + | + |  |  | + |  |  | + |  | + | + |  |  | + |  |  |  |  |  | + |  |  |  |  |  |  |
| NSW | SAPWH64 | _+_ |  |  | _+_ |  |  | _+_ |  |  | + | + |  |  | + |  |  | + |  | + | + |  |  | + |  |  |  |  |  | + | w |  |  |  |  |  |
| NSW | SAPWH94 | _+_ |  |  | _+_ |  |  | _+_ |  |  | + | + |  |  | + |  |  | + |  | + | + |  | + | + |  |  |  |  |  | + | + |  |  |  |  |  |
| NSW | SAPWH71 |  |  |  |  |  |  | _+_ |  |  | + | + |  |  | + |  |  | + |  | + | + |  |  | + |  |  |  |  |  | + |  |  |  |  |  |  |
| NSW | SAPCRGH95 | _+_ |  |  | _+_ |  |  | _+_ |  |  | + | + |  |  | + |  |  | + |  | + | + |  |  | + |  |  |  |  |  | + |  |  |  |  |  |  |
| NSW | SAPRPAH21 | _+_ |  |  | _+_ |  |  | _+_ |  |  | + | + |  |  | + |  |  | + |  | + | + |  |  | + |  |  |  |  |  | + | w |  |  |  |  |  |
| NSW | SAPRPAH7 | _+_ |  |  | _+_ |  |  | _+_ |  |  | + | + |  |  | + |  |  | + |  | + | + |  | + | + |  |  |  |  |  | + | + |  |  |  |  |  |
| NSW | SAPWH10 | _+_ |  |  | _+_ |  |  | _+_ |  |  | + | + |  |  | + |  |  | + |  | + | + |  | + | + |  |  |  |  |  | + | + |  |  |  |  |  |
| NSW | SAPWH53 | _+_ |  |  | _+_ |  |  | _+_ |  |  | + | + |  |  | + |  |  | + |  | + | + |  |  | + |  |  |  |  |  | + |  |  |  |  |  |  |
| NT | SAPRDH61 | _+_ |  |  | _+_ |  |  | _+_ |  |  | + | + |  |  | + |  |  | + |  | + | + |  |  | + |  |  |  |  |  | + |  |  |  |  |  |  |
| NT | SAPRDH27 | _+_ |  |  | _+_ |  |  | _+_ |  |  | + | + |  |  | + |  |  | + |  | + | + |  |  | + |  |  |  |  |  | + |  |  |  |  |  |  |
| NT | SAPRDH2 | _+_ |  |  | _+_ |  |  | _+_ |  |  | + | + |  |  | + |  |  | + |  | + | + |  |  | + |  |  |  |  |  | + | + |  |  |  |  |  |
| Qld | SAPRBH98 | _+_ |  |  | _+_ |  |  | _+_ |  |  | + | + |  |  | + |  |  | + |  | + | + |  | w | + |  |  |  |  |  | + | + |  |  |  |  |  |
| Qld | SAPRBH12 | _+_ |  |  | _+_ |  |  | _+_ |  |  | + | + |  |  | + |  |  | + |  | + | + |  |  | + |  |  |  |  |  | + |  |  |  |  |  |  |
| Qld | SAPGCH3 | _+_ |  |  | _+_ |  |  | _+_ |  |  | + | + |  |  | + |  |  | + |  | + | + |  |  | + |  |  |  |  |  | + |  |  |  |  |  |  |
| Qld | SAPRBH14 | _+_ |  |  | _+_ |  |  | _+_ |  |  | + | + |  |  | + |  |  | + |  | + | + |  |  | + |  |  |  |  |  | + |  |  |  |  |  |  |
| Qld | SAPCBH10 | _+_ |  |  | _+_ |  |  | _+_ |  |  | + | + |  |  | + |  |  | + |  | + | + |  | w | + |  |  |  |  |  | + | + |  |  |  |  |  |
| Qld | SAPGCH28 | _+_ |  |  | _+_ |  |  | _+_ |  |  | + | + |  |  | + |  |  | + |  | + | + |  |  | + |  |  |  |  |  | + | + |  |  |  |  |  |
| Qld | SAPRBH1 | _+_ |  |  | _+_ |  |  | _+_ |  |  | + | + |  |  | + |  |  | + |  | + | + |  |  | + |  |  |  |  |  | + | + |  |  |  |  |  |
| SA | SAPGPSA73 | _+_ |  |  | _+_ |  |  | _+_ |  |  | + | + |  |  | + |  |  | + |  | + | + |  | w | + |  |  |  |  |  | + | w |  |  |  |  |  |
| SA | SAPIMVS24 | _+_ |  |  | _+_ |  |  | _+_ |  |  | + | + |  |  | + |  |  | + |  | + | + |  |  | + |  |  |  |  |  | + | w |  |  |  |  |  |
| SA | SAPIMVS31 | _+_ |  |  | _+_ |  |  | _+_ |  |  | + | + |  |  | + |  |  | + |  | + | + |  | w | + |  |  |  |  |  | + | + |  |  |  |  |  |
| Vic | RCH74 | _+_ |  |  | _+_ |  |  | _+_ |  |  | + | + |  |  | + |  |  | + |  | + | + |  | + | + |  |  |  |  |  | + | + |  |  |  |  |  |
| Vic | SAPAH21 | _+_ |  |  | _+_ |  |  | _+_ |  |  | + | + |  |  | + |  |  | + |  | + | + |  |  | + |  |  |  |  |  | + | w |  |  |  |  |  |
| WA | 16790 | _+_ |  |  | _+_ |  |  | _+_ |  |  | + | + |  |  | + |  |  | + |  | + | + |  | + | + |  |  |  |  |  | + | + |  |  |  |  |  |
| WA | 16815 | _+_ |  |  | _+_ |  | w | _+_ |  |  | + | + |  |  | + |  |  | + |  | + | + |  | + | + |  |  |  |  |  | + | + |  |  |  |  |  |
| WA | 15586 | _+_ |  |  | _+_ |  |  | _+_ |  |  | + | + |  |  | + |  |  | + |  | + | + |  |  | + |  |  |  |  |  | + |  |  |  |  |  |  |
| WA | 15587 | _+_ |  |  | _+_ |  |  | _+_ |  |  | + | + |  |  | + |  |  | + |  | + | + |  |  | + |  |  |  |  |  | + |  |  |  |  |  |  |
| WA | 16414 | _+_ |  |  | _+_ |  |  | _+_ |  |  | + | + |  |  | + |  |  | + |  | + | + |  |  | + |  |  |  |  |  | + |  |  |  |  |  |  |
| WA | 16475 | _+_ |  |  | _+_ |  |  | _+_ |  |  | + | + |  |  | + |  |  | + |  | + | + |  |  | + |  |  |  |  |  | + | w |  |  |  |  |  |
| WA | 17164 | _+_ |  |  | _+_ |  | w | _+_ |  |  | + | + |  |  | + |  |  | + |  | + | + |  | + | + |  |  |  |  |  | + | + |  |  |  |  |  |
| WA | 18158 | _+_ |  |  | _+_ |  | w | _+_ |  |  | + | + |  |  | + |  |  | + |  | + | + |  |  | + |  |  |  |  |  | + | + |  |  |  |  |  |
| WA | 18385 | _+_ |  |  | _+_ |  |  | _+_ |  |  | + | + |  |  | + |  |  | + |  | + | + |  |  | + |  |  |  |  |  | + |  |  |  |  |  |  |
| WA | 18418 | _+_ |  |  | _+_ |  |  | _+_ |  |  | + | + |  |  | + |  |  | + |  | + | + |  | w | + |  |  |  |  |  | + | + |  |  |  |  |  |
| WA | 20198 | _+_ |  |  | _+_ |  |  | _+_ |  |  | + | + |  |  | + |  |  | + |  | + | + |  | w | + |  |  |  |  |  | + | + |  |  |  |  |  |
| WA | SAPRPH48 | _+_ |  |  | _+_ |  |  | _+_ |  |  | + | + |  |  | + |  |  | + |  | + | + |  | + | + |  |  |  |  |  | + | + |  |  |  |  |  |
| WA | 16908 | _+_ | + |  | _+_ |  | w | _+_ |  |  | + | + | + | w | + |  | + | + |  | + | + |  | + | + |  |  |  |  |  | + | + |  |  |  |  |  |
| WA | 17090 | _+_ |  |  | _+_ |  |  | _+_ |  |  | + | + |  |  | + |  |  | + |  | + | + |  | + | + |  |  |  |  |  | + | + |  |  |  |  |  |
| WA | 17195 | _+_ |  |  | _+_ |  | w | _+_ |  |  | + | + |  |  | + |  |  | + |  | + | + |  | w | + |  |  |  |  |  | + | + |  |  |  |  |  |
| WA | 20548 | _+_ |  |  | _+_ |  |  | _+_ |  |  | + | + |  |  | + |  |  | + |  | + | + |  |  | + |  |  |  |  |  | + | w |  |  |  |  |  |
| **Control Strain** | | | | | | | | | | | | | | | | | | | | | | | | | | | | | | | | | | | | |
| Vic | JKD6159 | _+_ |  |  | _+_ |  |  | _+_ |  |  | + | + |  |  | + |  |  | + |  | + | + |  |  | + |  |  |  |  |  | + | + |  |  |  |  |  |

Regions: ACT, Australian Capital Territory; NSW, New South Wales; NT, Northern Territory, Qld, Queensland; SA, South Australia; Vic, Victoria; WA, Western Australia

*bbp*, bone sialoprotein-binding protein gene (alleles); *clfA*, clumping factor A gene (alleles); *clfB*, clumping factor B gene (alleles); *cna*, collagen-binding adhesion gene; *ebh*, cell wall associated fibronectin-binding protein gene; *eno*, enolase gene; *fib*, fibrinogen binding protein gene (alleles); *ebpS*, cell surface elastin binding protein gene (alleles); *fnbA*, fibronectin-binding protein A gene (alleles); *fnbB*, fibronectin-binding protein B gene (alleles)

+, gene detected; w, gene detected but yielding weak or ambiguous signals

| **Supplementary Table 4: Microarray DNA ST93 MSCRAMMs and adhesion profile cont** | | | | | | | | | | | | | | | | | | | | | | | | |
| --- | --- | --- | --- | --- | --- | --- | --- | --- | --- | --- | --- | --- | --- | --- | --- | --- | --- | --- | --- | --- | --- | --- | --- | --- |
| **Region** | **Reference**  **Number** | **map** | **map (RF122)** | **map (MRSA252)** | **map (Mu50/MW2)** | **sdrC (all)** | **sdrC (B1)** | **sdrC (COL)** | **sdrC (Mu50)** | **sdrC (MW2/MRSA252/RF122)** | **sdrc (other than MRSA252/RF122)** | **sdrD (COL/MW2)** | **sdrD (Mu50)** | **sdrD (other)** | **sdrD (other than MRSA252/RF122)** | **vwb (all)** | **vwb (COL/MW2)** | **vwb (MRSA252)** | **vwb (Mu50)** | **vwb (RF122)** | **sasG** | **sasG (COL/Mu50)** | **sasG (MW2)** | **sasG (Other than MRSA252/RF122)** |
| **ST93 MSSA** | | | | | | | | | | | | | | | | | | | | | | | | |
| NT | WBG7735 |  |  |  |  | + |  | + |  |  |  |  |  | + | + | + |  |  |  |  |  |  |  |  |
| NT | WBG7762 |  |  |  |  | + |  | + |  |  |  |  |  | + | + | + |  |  |  |  |  |  |  |  |
| Qld | UQ40 |  |  |  |  | + |  | + |  |  |  |  |  | + | + | + |  |  |  |  |  |  |  |  |
| Vic | DP2039 |  |  |  |  | + |  | + |  |  |  |  |  | + | + | + |  |  |  |  |  |  |  |  |
| WA | C229T |  |  |  |  | + |  | + |  |  |  |  |  | + | + | + |  |  |  |  |  |  |  |  |
| WA | N126W |  |  |  |  | + |  | + |  |  |  |  |  | + | + | + |  |  |  |  |  |  |  |  |
| WA | W17S |  |  |  |  | + |  | + |  |  |  |  |  | + | + | + |  |  |  |  |  |  |  |  |
| WA | Y113S |  |  |  |  | + |  | + |  |  |  |  |  | + | + | + |  |  |  |  |  |  |  |  |
| WA | 9506160A |  |  |  |  | + |  | + |  |  |  |  |  | + | + | + |  |  |  |  |  |  |  |  |
| WA | 9509712N |  |  |  |  | + |  | + |  |  |  |  |  | + | + | + |  |  |  |  |  |  |  |  |
| WA | 9524093R |  |  |  |  | + |  | + |  |  |  |  |  | + | + | + |  |  |  |  |  |  |  |  |
| WA | 9525206A |  |  |  |  | + |  | + |  |  |  |  |  | + | + | + |  |  |  |  |  |  |  |  |
| WA | 9529120L |  |  |  |  | + |  | + |  |  |  |  |  | + | + | + |  |  |  |  |  |  |  |  |
| **ST93 MSSA** | | | | | | | | | | | | | | | | | | | | | | | | |
| ACT | SAPTCH92 |  |  |  |  | + |  | + |  |  |  |  |  | + | + | + |  |  |  |  |  |  |  |  |
| ACT | SAPTCH53 |  |  |  |  | + |  | + |  |  |  |  |  | + | + | + |  |  |  |  |  |  |  |  |
| NSW | SAPRPAH96 |  |  |  |  | + |  | + |  |  |  |  |  | + | + | + |  |  |  |  |  |  |  |  |
| NSW | SAPWH23 |  |  |  |  | + |  | + |  |  |  |  |  | + |  | + |  |  |  |  |  |  |  |  |
| NSW | SAPWH39 |  |  |  |  | + |  | + |  |  |  |  |  | + | + | + |  |  |  |  |  |  |  |  |
| NSW | SAPWH61 |  |  |  |  | + |  | + |  |  |  |  |  | + | + | + |  |  |  |  |  |  |  |  |
| NSW | SAPWH64 |  |  |  |  | + |  | + |  |  |  |  |  | + | + | + |  |  |  |  |  |  |  |  |
| NSW | SAPWH94 |  |  |  |  | + |  | + |  |  |  |  |  | + | + | + |  |  |  |  |  |  |  |  |
| NSW | SAPWH71 |  |  |  |  | + |  | + |  |  |  |  |  | + |  | + |  |  |  |  |  |  |  |  |
| NSW | SAPCRGH95 |  |  |  |  | + |  | + |  |  |  |  |  | + |  | + |  |  |  |  |  |  |  |  |
| NSW | SAPRPAH21 |  |  |  |  | + |  | + |  |  |  |  |  | + | + | + |  |  |  |  |  |  |  |  |
| NSW | SAPRPAH7 |  |  |  |  | + |  | + |  |  |  |  |  | + | + | + |  |  |  |  |  |  |  |  |
| NSW | SAPWH10 |  |  |  |  | + |  | + |  |  |  |  |  | + | + | + |  |  |  |  |  |  |  |  |
| NSW | SAPWH53 |  |  |  |  | + |  | + |  |  |  |  |  | + | + | + |  |  |  |  |  |  |  |  |
| NT | SAPRDH61 |  |  |  |  | + |  | + |  |  |  |  |  | + | + | + |  |  |  |  |  |  |  |  |
| NT | SAPRDH27 |  |  |  |  | + |  | + |  |  |  |  |  | + | + | + |  |  |  |  |  |  |  |  |
| NT | SAPRDH2 |  |  |  |  | + |  | + |  |  |  |  |  | + | + | + |  |  |  |  |  |  |  |  |
| Qld | SAPRBH98 |  |  |  |  | + |  | + |  |  |  |  |  | + | + | + |  |  |  |  |  |  |  |  |
| Qld | SAPRBH12 |  |  |  |  | + |  | + |  |  |  |  |  | + |  | + |  |  |  |  |  |  |  |  |
| Qld | SAPGCH3 |  |  |  |  | + |  | + |  |  |  |  |  | + | + | + |  |  |  |  |  |  |  |  |
| Qld | SAPRBH14 |  |  |  |  | + |  | + |  |  |  |  |  | + | + | + |  |  |  |  |  |  |  |  |
| Qld | SAPCBH10 |  |  |  |  | + |  | + |  |  |  |  |  | + | + | + |  |  |  |  |  |  |  |  |
| Qld | SAPGCH28 |  |  |  |  | + |  | + |  |  |  |  |  | + | + | + |  |  |  |  |  |  |  |  |
| Qld | SAPRBH1 |  |  |  |  | + |  | + |  |  |  |  |  | + | + | + |  |  |  |  |  |  |  |  |
| SA | SAPGPSA73 |  |  |  |  | + |  | + |  |  |  |  |  | + | + | + |  |  |  |  |  |  |  |  |
| SA | SAPIMVS24 |  |  |  |  | + |  | + |  |  |  |  |  | + | + | + |  |  |  |  |  |  |  |  |
| SA | SAPIMVS31 |  |  |  |  | + |  | + |  |  |  |  |  | + | + | + |  |  |  |  |  |  |  |  |
| Vic | SAPRCH74 |  |  |  |  | + |  | + |  |  |  |  |  | + | + | + |  |  |  |  |  |  |  |  |
| Vic | SAPAH21 |  |  |  |  | + |  | + |  |  |  |  |  | + | + | + |  |  |  |  |  |  |  |  |
| WA | 16790 |  |  |  |  | + |  | + |  |  |  |  |  | + | + | + |  |  |  |  |  |  |  |  |
| WA | 16815 |  |  |  |  | + |  | + |  |  |  |  |  | + | + | + |  |  |  |  |  |  |  |  |
| WA | 15586 |  |  |  |  | + |  | + |  |  |  |  |  | + | + | + |  |  |  |  |  |  |  |  |
| WA | 15587 |  |  |  |  | + |  | + |  |  |  |  |  | + | + | + |  |  |  |  |  |  |  |  |
| WA | 16414 |  |  |  |  | + |  | + |  |  |  |  |  | + | + | + |  |  |  |  |  |  |  |  |
| WA | 16475 |  |  |  |  | + |  | + |  |  |  |  |  | + | + | + |  |  |  |  |  |  |  |  |
| WA | 17164 |  |  |  |  | + |  | + |  |  |  |  |  | + | + | + |  |  |  |  |  |  |  |  |
| WA | 18158 |  |  |  |  | + |  | + |  |  |  |  |  | + | + | + |  |  |  |  |  |  |  |  |
| WA | 18385 |  |  |  |  | + |  | + |  |  |  |  |  | + | + | + |  |  |  |  |  |  |  |  |
| WA | 18418 |  |  |  |  | + |  | + |  |  |  |  |  | + | + | + |  |  |  |  |  |  |  |  |
| WA | 20198 |  |  |  |  | + |  | + |  |  |  |  |  | + | + | + |  |  |  |  |  |  |  |  |
| WA | SAPRPH48 |  |  |  |  | + |  | + |  |  |  |  |  | + | + | + |  |  |  |  |  |  |  |  |
| WA | 16908 |  |  |  |  | + |  | + | w | w |  |  |  | + | + | + |  |  |  |  |  |  |  |  |
| WA | 17090 |  |  |  |  | + |  | + |  |  |  |  |  | + | + | + |  |  |  |  |  |  |  |  |
| WA | 17195 |  |  |  |  | + |  | + |  |  |  |  |  | + | + | + |  |  |  |  |  |  |  |  |
| WA | 20548 |  |  |  |  | + |  | + |  |  |  |  |  | + | + | + |  |  |  |  |  |  |  |  |
| Vic | JK06159 |  |  |  |  | + |  | + |  |  |  |  |  | + | + | + |  |  |  |  |  |  |  |  |

Regions: ACT, Australian Capital Territory; NSW, New South Wales; NT, Northern Territory, Qld, Queensland; SA, South Australia; Vic, Victoria; WA, Western Australia

*map*, major histocompatability complex class II analogue protein gene (alleles); *sdrC*, ser-asp rich fibrinogen-/bone sialoprotein-binding protein C gene (alleles); *sdrD*, ser-asp rich fibrinogen-/bone sialoprotein-binding protein D gene (alleles);*vwb*, van Willebrand factor binding protein gene (allele); *sasG*, *Staphylococcus aureus* surface protein G gene (alleles)

+, gene detected; w, gene detected but yielding weak or ambiguous signals
