# Supplementary material for: The Molecular Epidemiology of the Highly Virulent ST93 Australian Community Staphylococcus aureus Strain
Source: PLoS One. 2012 Aug 10;7(8):e43037. doi: 10.1371/journal.pone.0043037 (PMC3416834; doi:10.1371/journal.pone.0043037)
Supplement: Table S5 — Microarray DNA ST93 immunevasion and miscellaneous profile. (DOCX) [file pone.0043037.s004.docx]

| **Supplementary Table 5: Microarray DNA ST93 immunevasion and miscellaneous profile** | | | | | | | | | | | | | | | | | | | | | | | | | | | | | | | | | | |
| --- | --- | --- | --- | --- | --- | --- | --- | --- | --- | --- | --- | --- | --- | --- | --- | --- | --- | --- | --- | --- | --- | --- | --- | --- | --- | --- | --- | --- | --- | --- | --- | --- | --- | --- |
| **Region** | **Reference**  **Number** | **isaB** | **isaB (MRSA252)** | **mprF** | **mprF (COL/MW2)** | **mprF (Mu50/MRSA252)** | **isdA** | **isdA (MRSA252)** | **isdA (Other than MRSA252)** | **ImrP** | **ImrP (RF122)** | **Q2YUB3** | **hsdS1 (RF122)** | **hsdS2 (ST5/ST8)** | **hsdS2 (MW2/476)** | **hsdS2 (RF122)** | **hsdS2 (MRSA252)** | **hsdS3 (other than RF122/MRSA252)** | **hsdS3 (ST8/ST1/RF122)** | **hsdS3 (Mu50/N315)** | **hsdS3 (CC51/MRSA252)** | **hsdS3 (MRSA252)** | **hsdSx (CC25)** | **hsdSx (CC15)** | **hsdSx (etd)** | **Q2FXCO** | **Q7A4X2** | **hysA1 (MRSA252)** | **hysA1 (MRSA252/RF122)/hysA2** | **hysA1 (MRSA252/RF122)/hysA2**  **(COL/USA300)** | **hysA2 (all except MRSA252)** | **hysA2 (COL/USA300/NCTC)** | **hysA2 ( all except COL/USA300/NCTC)** | **hysA2 (MRSA252)** |
| **ST93 MSSA** | | | | | | | | | | | | | | | | | | | | | | | | | | | | | | | | | | |
| NT | WBG7735 | + | + |  |  |  | + |  | + | + |  |  |  |  |  |  |  |  |  |  |  | + |  |  |  |  | + |  | + | + |  | + |  |  |
| NT | WBG7762 | + | + |  |  |  | + |  | + | + |  |  |  |  |  |  |  |  |  |  |  | + |  |  |  |  | + |  | + | + |  | + |  |  |
| Qld | UQ40 | + | + |  |  |  | + |  | + | + |  |  |  |  |  |  |  |  |  |  |  | + |  |  |  |  | + |  | + | + |  | + |  | w |
| Vic | DP2039 | + | + | w |  | w | + | w | + | + |  | w |  |  | w |  |  |  |  |  |  | + | w | w |  |  | + |  | + | + |  | + |  | + |
| WA | C229T | + | + |  |  |  | + |  | + | + |  |  |  |  | + |  |  |  |  |  |  | + |  |  |  |  | + |  | + | + |  | + |  |  |
| WA | N126W | + | + |  |  |  | + |  | + | + |  |  |  |  |  |  |  |  |  |  |  | + |  |  |  |  | + |  | + | + |  | + |  |  |
| WA | W17S | + | + | w |  | w | + |  | + | + |  | w |  |  |  |  |  |  |  |  |  | + | w |  |  |  | + |  | + | + |  | + |  | w |
| WA | Y113S | + | + |  |  |  | + |  | + | + |  |  |  |  | + |  |  |  |  |  |  | + |  |  |  |  | + |  | + | + |  | + |  |  |
| WA | 9506160A | + | + |  |  |  | + |  | + | + |  |  |  |  | + |  |  |  |  |  |  | + |  |  |  |  | + |  | + | + |  | + |  | + |
| WA | 9509712N | + | + |  |  |  | + |  | + | + |  |  |  |  | + |  |  |  |  |  |  | + |  |  |  |  | + |  | + | + |  | + |  |  |
| WA | 9524093R | + | + |  |  |  | + |  | + | + |  |  |  |  | + |  |  |  |  |  |  | + |  |  |  |  | + |  | + | + |  | + |  |  |
| WA | 9525206A | + | + |  |  |  | + |  | + | + |  |  |  |  |  |  |  |  |  |  |  | + |  |  |  |  | + |  | + | + |  | + |  |  |
| WA | 9529120L | + | + |  |  |  | + |  | + | + |  |  |  |  | + |  |  |  |  |  |  | + |  |  |  |  | + |  | + | + |  | + |  |  |
| **ST93 MRSA** | | | | | | | | | | | | | | | | | | | | | | | | | | | | | | | | | | |
| ACT | SAPTCH92 | + | + |  |  |  | + |  | + | + |  |  |  |  | + |  |  |  |  |  |  | + |  |  |  |  | + |  | + | + |  | + |  | + |
| ACT | SAPTCH53 | + | + |  |  |  | + |  | + | + |  |  |  |  | + |  |  |  |  |  |  | + |  |  |  |  | + |  | + | + |  | + |  | w |
| NSW | SAPRPAH96 | + | + |  |  |  | + |  | + | + |  |  |  |  |  |  |  |  |  |  |  | + |  |  |  |  | + |  | + | + |  | + |  |  |
| NSW | SAPWH23 | + | + |  |  |  | + |  | + | + |  |  |  |  |  |  |  |  |  |  |  | + |  |  |  |  | + |  | w | + |  | + |  |  |
| NSW | SAPWH39 | + | + |  |  |  | + |  | + | + |  |  |  |  |  |  |  |  |  |  |  | + |  |  |  |  | + |  | + | + |  | + |  | w |
| NSW | SAPWH61 | + | + |  |  |  | + |  | + | + |  |  |  |  |  |  |  |  |  |  |  | + |  |  |  |  | + |  | w | + |  | + |  |  |
| NSW | SAPWH64 | + | + |  |  |  | + |  | + | + |  |  |  |  |  |  |  |  |  |  |  | + |  |  |  |  | + |  | + | + |  | + |  |  |
| NSW | SAPWH94 | + | + |  |  |  | + |  | + | + |  |  |  |  |  |  |  |  |  |  |  | + |  |  |  |  | + |  | + | + |  | + |  | w |
| NSW | SAPWH71 | + | + |  |  |  | + |  | + | + |  |  |  |  |  |  |  |  |  |  |  | + |  |  |  |  | + |  | + | + |  | + |  |  |
| NSW | SAPCRGH95 | + | + |  |  |  | + |  | + | + |  |  |  |  |  |  |  |  |  |  |  | + |  |  |  |  | + |  |  | + |  | + |  |  |
| NSW | SAPRPAH21 | + | + |  |  |  | + |  | + | + |  |  |  |  |  |  |  |  |  |  |  | + |  |  |  |  | + |  | + | + |  | + |  |  |
| NSW | SAPRPAH7 | + | + |  |  |  | + |  | + | + |  |  |  |  | + |  |  |  |  |  |  | + |  |  |  |  | + |  | + | + |  | + |  | + |
| NSW | SAPWH10 | + | + |  |  |  | + |  | + | + |  |  |  |  | w |  |  |  |  |  |  | + |  |  |  |  | + |  | + | + |  | + |  | + |
| NSW | SAPWH53 | + | + |  |  |  | + |  | + | + |  |  |  |  |  |  |  |  |  |  |  | + |  |  |  |  | + |  | + | + |  | + |  |  |
| NT | SAPRDH61 | + | + |  |  |  | + |  | + | + |  |  |  |  |  |  |  |  |  |  |  | + |  |  |  |  | + |  | + | + |  | + |  |  |
| NT | SAPRDH27 | + | + |  |  |  | + |  | + | + |  |  |  |  |  |  |  |  |  |  |  | + |  |  |  |  | + |  | + | + |  | + |  |  |
| NT | SAPRDH2 | + | + |  |  |  | + |  | + | + |  |  |  |  |  |  |  |  |  |  |  | + |  |  |  |  | + |  | + | + |  | + |  |  |
| Qld | SAPRBH98 | + | + |  |  |  | + |  | + | + |  |  |  |  | + |  |  |  |  |  |  | + |  |  |  |  | + |  | + | + |  | + |  | w |
| Qld | SAPRBH12 | + | + |  |  |  | + |  | + | + |  |  |  |  |  |  |  |  |  |  |  | + |  |  |  |  | + |  |  | + |  | + |  |  |
| Qld | SAPGCH3 | + | + |  |  |  | + |  | + | + |  |  |  |  |  |  |  |  |  |  |  | + |  |  |  |  | + |  | + | + |  | + |  |  |
| Qld | SAPRBH14 | + | + |  |  |  | + |  | + | + |  |  |  |  |  |  |  |  |  |  |  | + |  |  |  |  | + |  | w | + |  | + |  |  |
| Qld | SAPCBH10 | + | + |  |  |  | + |  | + | + |  |  |  |  | + |  |  |  |  |  |  | + |  |  |  |  | + |  | + | + |  | + |  | w |
| Qld | SAPGCH28 | + | + |  |  |  | + |  | + | + |  |  |  |  | + |  |  |  |  |  |  | + |  |  |  |  | + |  | + | + |  | + |  |  |
| Qld | SAPRBH1 | + | + |  |  |  | + |  | + | + |  |  |  |  | w |  |  |  |  |  |  | + |  |  |  |  | + |  | + | + |  | + |  |  |
| SA | SAPGPSA73 | + | + |  |  |  | + |  | + | + |  |  |  |  | w |  |  |  |  |  |  | + |  |  |  |  | + |  | + | + |  | + |  |  |
| SA | SAPIMVS24 | + | + |  |  |  | + |  | + | + |  |  |  |  |  |  |  |  |  |  |  | + |  |  |  |  | + |  | + | + |  | + |  |  |
| SA | SAPIMVS31 | + | + |  |  |  | + |  | + | + |  |  |  |  | w |  |  |  |  |  |  | + |  |  |  |  | + |  | + | + |  | + |  |  |
| Vic | SAPRCH74 | + | + |  |  |  | + |  | + | + |  |  |  |  | + |  |  |  |  |  |  | + |  |  |  |  | + |  | + | + |  | + |  | w |
| Vic | SAPAH21 | + | + |  |  |  | + |  | + | + |  |  |  |  | w |  |  |  |  |  |  | + |  |  |  |  | + |  | + | + |  | + |  |  |
| WA | 16790 | + | + |  |  |  | + |  | + | + |  |  |  |  | + |  |  |  |  |  |  | + |  |  |  |  | + |  | + | + |  | + |  | w |
| WA | 16815 | + | + | + |  | + | + | w | + | + |  | + |  |  | w |  |  |  |  |  |  | + | + | w | + |  | + |  | + | + |  | + |  | + |
| WA | 15586 | + | + |  |  |  | + |  | + | + |  |  |  |  |  |  |  |  |  |  |  | + |  |  |  |  | + |  | + | + |  | + |  |  |
| WA | 15587 | + | + |  |  |  | + |  | + | + |  |  |  |  | + |  |  |  |  |  |  | + |  |  |  |  | + |  | + | + |  | + |  | w |
| WA | 16414 | + | + |  |  |  | + |  | + | + |  |  |  |  |  |  |  |  |  |  |  | + |  |  |  |  | + |  | + | + |  | + |  |  |
| WA | 16475 | + | + | w |  | w | + |  | + | + |  |  |  |  | w |  |  |  |  |  |  | + | w |  |  |  | + |  | + | + |  | + |  | w |
| WA | 17164 | + | + | + |  | + | + | w | + | + |  | + |  |  | w |  |  |  |  |  |  | + | + | w |  |  | + |  | + | + |  | + |  | + |
| WA | 18158 | + | + |  |  |  | + |  | + | + |  | w |  |  | + |  |  |  |  |  |  | + | w | w |  |  | + |  | + | + |  | + |  | w |
| WA | 18385 | + | + |  |  |  | + |  | + | + |  |  |  |  |  |  |  |  |  |  |  | + |  |  |  |  | + |  | + | + |  | + |  |  |
| WA | 18418 | + | + | w |  | w | + |  | + | + |  |  |  |  |  |  |  |  |  |  |  | + |  |  |  |  | + |  | + | + |  | + |  | w |
| WA | 20198 | + | + |  |  |  | + |  | + | + |  |  |  |  |  |  |  |  |  |  |  | + |  |  |  |  | + |  | + | + |  | + |  |  |
| WA | SAPRPH48 | + | + | w |  | w | + |  | + | + |  |  |  |  |  |  |  |  |  |  |  | + | w |  |  |  | + |  | + | + |  | + |  | + |
| WA | 16908 | + | + | + | w | + | + | + | + | + |  | + |  |  | w |  |  |  |  |  |  | + | + | w |  |  | + |  | + | + |  | + |  | + |
| WA | 17090 | + | + |  |  |  | + |  | + | + |  | w |  |  |  |  |  |  |  |  |  | + |  |  |  |  | + |  | + | + |  | + |  | w |
| WA | 17195 | + | + | w |  | w | + |  | + | + |  | w |  |  |  |  |  |  |  |  |  | + | w | w |  |  | + |  | + | + |  | + |  | w |
| WA | 20548 | + | + |  |  |  | + |  | + | + |  |  |  |  |  |  |  |  |  |  |  | + |  |  |  |  | + |  | + | + |  | + |  |  |
| **Control Strain** | | | | | | | | | | | | | | | | | | | | | | | | | | | | | | | | | | |
| Vic | JK06159 | + | + |  |  |  | + |  | + | + |  |  |  |  | + |  |  |  |  |  |  | + |  |  |  |  | + |  | + | + |  | + |  |  |

Regions: ACT, Australian Capital Territory; NSW, New South Wales; NT, Northern Territory, Qld, Queensland; SA, South Australia; Vic, Victoria; WA, Western Australia

*isaB*, immunodominant antigen B gene (alleles); *mprF*, defensin resistance gene protein gene (alleles); *isdA*, transferrin-binding protein gene (alleles); *ImrP*, hypothetical protein, similar to integral membrane protein LmrP gene (alleles); *Q2YUB3*, Unspecific efflux/transporter gene; hsdS1, type 1 site-specific deoxyribonuclease subunit, 1^st^ locus gene; *hsdS2*, type 1 site-specific deoxyribonuclease subunit, 2^nd^ locus gene (alleles); hsdS3, type 1 site-specific deoxyribonuclease subunit, 3^rd^ locus gene (alleles); hsdSx, type 1 site-specific deoxyribonuclease subunit, unknown locus gene (alleles); *Q2FXCO*, hypothetical protein gene, located next to serine protease operon; *Q7A4X2*, hypothetical protein gene; *hysA1*, hyaluronate lyase first locus gene (alleles); *hysA2*, hyaluronate lyase second locus gene (alleles)

+, gene detected; w, gene detected but yielding weak or ambiguous signals
